# Granular Hydrogels as Modular Biomaterials: From Structural Design to Biological Responses

**DOI:** 10.1002/adhm.202502462

**Published:** 2025-09-26

**Authors:** Asmasadat Vaziri, Renata Maia, Pei Zhang, Liliana Agresti, Jelmer Sjollema, Mohammad‐Ali Shahbazi, Hélder A. Santos

**Affiliations:** ^1^ Department of Biomaterials and Biomedical Technology The Personalized Medicine Research Institute (PRECISION) University Medical Center Groningen University of Groningen AV Groningen 9713 The Netherlands; ^2^ Drug Research Program Division of Pharmaceutical Chemistry and Technology Faculty of Pharmacy University of Helsinki Helsinki 00014 Finland; ^3^ Finncure Oy Lars Sonckin Kaari 14 Espoo 02600 Finland

**Keywords:** biomedical engineering, granular hydrogel, Inter/intraparticle crosslinking, microparticles, microporous

## Abstract

Over the past decade, granular hydrogels have been widely utilized as a cell‐free or cell‐laden platform to deliver therapeutics (e.g., cells and drugs) for tissue repair, or as a bioink or supporting bed for bioprinting. Owing to their inherent microporosity and modularity, various granular hydrogels with functional applications for in vivo use have been fabricated to enhance cell infiltration, spreading and migration, harness immune response, and promote tissue regeneration. In this review, an updated overview of the current state‐of‐the‐art is provided for granular hydrogel development, by highlighting the interplay between design parameters and structural characteristics, like porosity, microstructure, rheological behavior, injectability, and degradability, and their influence on biological responses in various biomedical engineering applications.

## Graphical abstract

Granular hydrogels are now emerging as promising biomaterials due to their inherent microporousity, injectability, and modularity. They have shown improvements in cell viability and migration, cellular/tissue infiltration, host tissue integration, mitigated foreign body response, and tissue regeneration. The latest works on granular hydrogels are reviewed, identifying key parameters to tailor their physicochemical properties, and highlighting their biological responses.

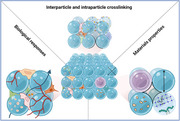

## 1 Introduction

Granular hydrogels are emerging as modular biomaterials, composed of micron‐scale particles (building blocks) that form a 3D porous hydrogel when interlinked.^[^ ^1^ ^]^ The idea of assembling cell‐embedded microparticles with the purpose of decoupling chemical and mechanical properties and cellular function was explored by Elbert's group in 2011. Polyethylene glycol (PEG) spherical microparticles were mixed with collagen І, and collected by centrifuge and formed a microporous scaffold, named “modugels”, crosslinked by the remaining functional groups on the surface of microspheres.^[^ ^2^ ^]^ In another example, Ghodousi et al. explored the microparticles’ assembly by adding them to a glass substrate in phosphate buffer saline, and by changing the hydrophilicity of surfaces and pouring off the excess solution, microparticles were partially trapped in the remaining buffer.^[^ ^3^ ^]^ An earlier example of microparticle assembly is the work reported by Sefton et al. in 2006, in which they produced cylinders by cutting a tubular collagen (COL) gel. The cylinders were coated with endothelial cells and assembled through the adhesion of cells, when subjected to blood flow.^[^ ^4^ ^]^

The inherent interconnected void space among microparticles is the most prominent feature of granular hydrogels, which enhances oxygen and nutrient transport, facilitating cell growth, migration, and proliferation both in vitro and in vivo.^[^ ^5^, ^6^ ^]^ Moreover, studies have shown that cell‐free granular hydrogels allow endogenous cell infiltration by mimicking the percolated interstitial spaces in native tissue, unlike nanoporous bulk hydrogels that often lead to a fibrotic foreign body response once placed in vivo.^[^ ^7^ ^]^ Thus, granular hydrogels can support vascularization and integration with host tissue, ultimately resulting in successful tissue regeneration.

**Figure 1:**
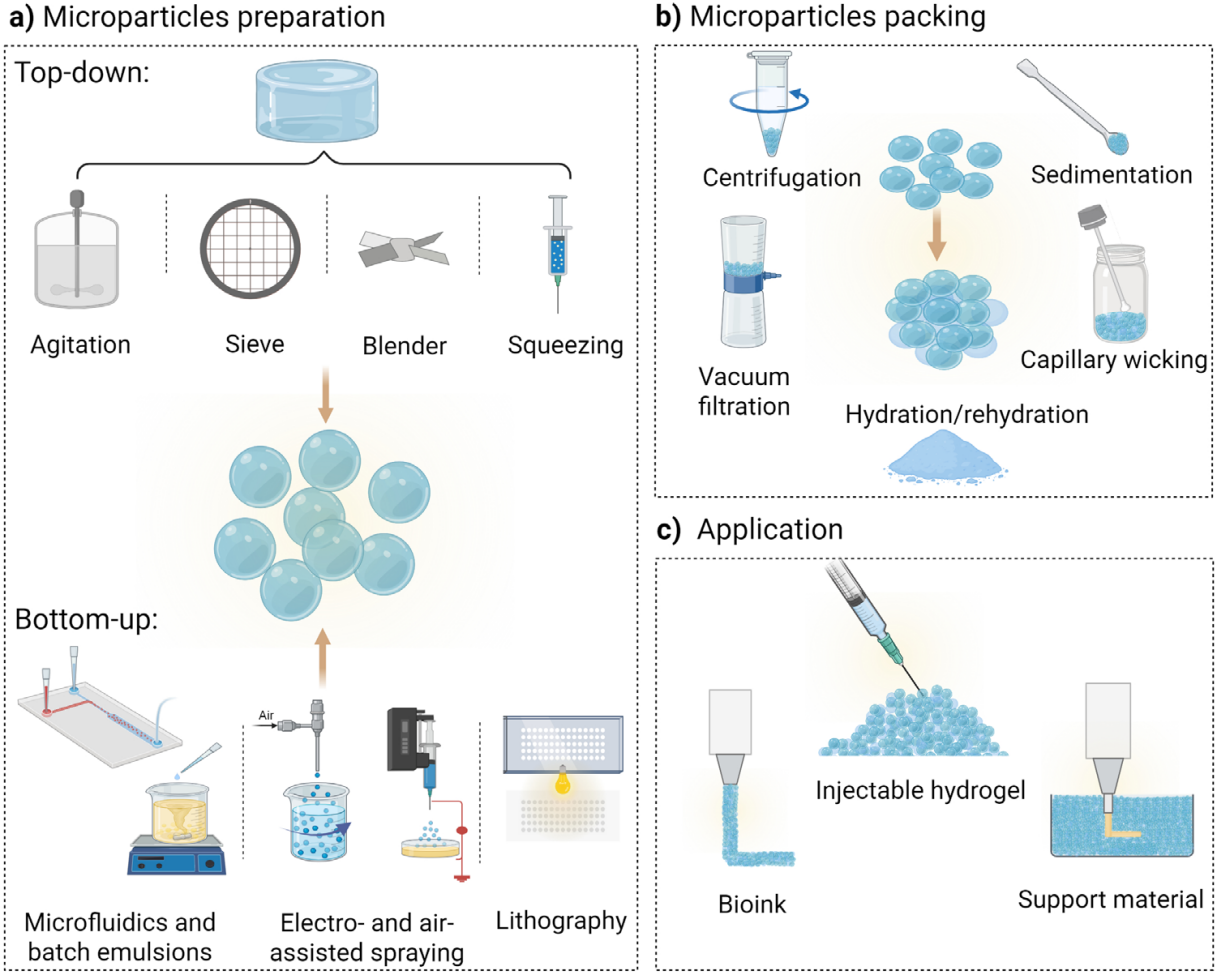
Overview of the main top‐down and bottom‐up microparticle fabrication techniques, their assembly in the form of granular hydrogels, and the desired applications. a) Microparticle fabrication techniques include: Top‐down approach using mechanical devices like agitator, metal sieves, blender, syringe, and nozzles of different sizes, where bulk hydrogel is fragmented into micro‐size particles of any shape. Bottom‐up approaches include microfluidic chip and batch emulsion technique, electrohydrodynamic spraying and extrusion devices, and lithography. b) Different assembly techniques that are now available for packing microparticles with desired packing density to realize a physical transition from suspension to a jammed state and form granular hydrogels, including centrifugation, sedimentation, and collection by spatula, vacuum filtration, capillary wicking, and hydration/dehydration process through lyophilization. c) Granular hydrogels have shown promise to be used as injectable hydrogels for minimally invasive treatments, for extrusion bioprinting, and as a supporting bed for embedded bioprinting. Created by BioRender.com.

Microparticles can be made from the same biomaterials as bulk hydrogels. A summary of common microparticle fabrication and assembly strategies, as well as their desired applications as a granular hydrogel/bioink is summarized in **Figure** **1**. Various studies have shown that the size, shape, topography, degradability, and mechanics of individual microparticles, as well as their packing mechanism and density, all influence the behavior of final granular hydrogel,^[^ ^8^, ^9^, ^10^, ^11^, ^12^, ^13^ ^]^ allowing a high level of modularity and flexibility to achieve desirable properties. In this regard, particle fraction is a critical factor, as it is inversely proportional to the void fraction. Void fractions can be modulated by modifying particle size, shape, packing, and particle fraction.^[^ ^14^, ^15^, ^16^ ^]^ For example, tightly packed microparticles generate smaller void fractions, whereas loosely packed microparticles create larger void fractions.^[^ ^17^ ^]^

Notably, the surface of microparticles can be functionalized with various bioactive agents, introduced as microporous annealed particles (MAP), to reinforce mechanical stability. Different types of microparticles can be mixed together to form a heterogeneous platform with multiple signals and functionalities.^[^ ^18^ ^]^ Given the capability to tune the structural and physicochemical properties of granular hydrogels for different purposes, more studies have recently attempted to recapitulate the tissue‐like heterogeneity within a single hydrogel. **Table** **1** summarizes the potential advantages of granular hydrogels over bulk hydrogels, including tunable porosity, extrudability, heterogeneity, better cellular function, and tissue maturation.

**Table 1:** Summary of advantages of granular hydrogels over conventional bulk hydrogels.

Hydrogel characteristics	Granular hydrogel	Bulk hydrogel
Porosity	Microporous; tuned by microparticle size, shape, and packing density to support cell functions	Nanoporous; limits mass transport and cell access
Injectability/Printability	Small size enables high injectability through small needles regardless of polymer concentration; ideal for extrusion bioprinting, due to the shear‐thinning behavior	Might need high extrusion forces that could harm cell viability; poor printability, often need high polymer concentration or thickeners
Polymer concentration and mechanical flexibility	High	Low
Heterogeneity	Multiple types of microparticles can be mixed to create diverse compartments	Relatively homogenous
Cellular function	Supports cell proliferation, differentiation and migration due to the interconnected micro pore space	Relative low cell viability and migration due to the limited diffusivity, which restricts oxygen and nutrients accessibility to cells
Tissue maturation	Supports angiogenesis and extracellular matrix (ECM) excretion	Relative low ECM production

So far, several review papers have discussed the concepts of microgels fabrication and assembly.^[^ ^19^, ^20^, ^21^, ^22^ ^]^ For example, a recent review in 2025 discussed the microgels’ intra‐ and inter‐particle crosslinking strategies, with a primary focus on the resulting characteristics of granular hydrogels.^[^ ^23^ ^]^ In another work, Charlet et al. have reviewed the effect of inter‐particle crosslinking on the mechanical properties of these materials.^[^ ^24^ ^]^ Emerging works on the granular hydrogels application have been highlighted elsewhere as an ink^[^ ^25^, ^26^ ^]^ or a supporting bath^[^ ^27^ ^]^ to develop biofabrication techniques.^[^ ^19^ ^]^ For example, Daly described how each microgel design parameter such as size, shape, and stiffness can influence bioprinting performance.^[^ ^19^ ^]^ The unique attributes of these materials have been also discussed in detail on the granular hydrogels’ immunomodulatory potential^[^ ^28^ ^]^ and tissue engineering, with an emphasis on cartilage,^[^ ^29^ ^]^ muscoskeletal,^[^ ^30^ ^]^ and endogenous tissue repair.^[^ ^31^ ^]^

**Figure 2:**
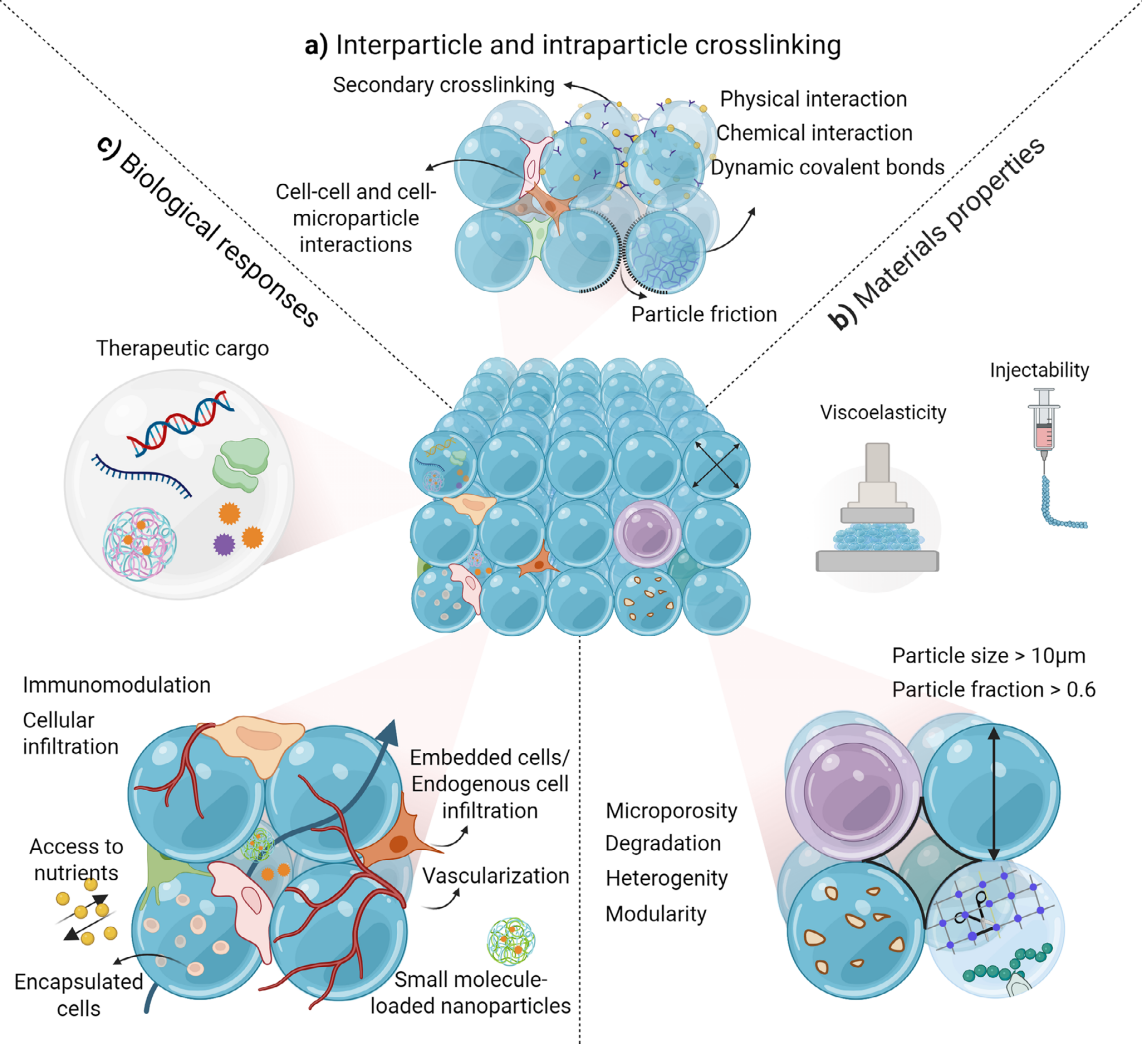
Designing parameters of biomimetic granular hydrogels of varied networks, materials characteristics, and biological performance for tissue repair and remodeling. a) Intraparticle interactions include physical, chemical, and dynamic covalent crosslinking chemistries used for microparticle fabrication. Interparticle interactions include surface particle frictions, secondary crosslinking, cell‐cell and cell‐microparticle interactions are corresponded for transition of microparticles suspension into a packed state to form a granular hydrogel. b) Materials characteristics provide multiple design parameters that can be utilized to create a programmed regenerative microenvironment through mimicking rheological properties, stiffness, and heterogenicity by mixing different microparticle species such as porous and core‐shell microparticles, with different geometries, varied µ‐scale porosity, and multifunctionality. c) The biological performance of granular hydrogels is affected by 3D microscale porosity similar to the micron size of cells, that can allow cellular infiltration and spreading, compartmentalization for loading multiple cells and small molecules, such as peptides.

Here, we provide a broad overview of the recent advances in the design of granular hydrogels with a focus on the various structural advancements that control the hydrogel's properties and their cellular functionalities. There is a significant diversity and interplay in the structural and physicochemical properties of granular hydrogels, which is a strength for their use as biomimetic matrices. While earlier reviews have addressed, to some extent, the principles of the design parameters, like the jamming transition and intra‐ and inter‐particle crosslinking networks, a comprehensive perspective on the structural and functional parameters remains limited. To simplify the interplay between design parameters, structural features and biological responses, we illustrated them in three categories, as shown in **Figure** **2**.

Filling the gap from existing reviews, we present here specific examples of where granular hydrogels have been used, both as cell‐free and cell‐embedded platforms. Special emphasis is placed on four different categories, comprising therapeutic cargo, in vitro cell culture, spheroid formation, and endogenous tissue regeneration. Finally, the challenges and limitations associated with the development of granular hydrogels with an outlook for future studies are also highlighted and discussed.

## 2 Microparticle Fabrication and Assembly

Granular hydrogels are composed of micro‐scale building blocks in the form of microparticles that are packed together through different assembly techniques.^[^ ^11^, ^32^, ^33^ ^]^ Microparticles can be prepared using bottom‐up strategies like microfluidics, water‐in‐oil batch emulsion, electrospraying, or top‐down strategies like mechanical fragmentation of bulk hydrogels (Figure 1a).^[^ ^34^, ^35^, ^36^, ^37^ ^]^ Depending on the microparticle formation methods, their microarchitecture, size, and size distribution can be tuned. Microparticles are made with the same chemistries and have similar properties as bulk hydrogels (**Table** **2**). Similar to bulk hydrogels,^[^ ^38^ ^]^ intra‐particle crosslinking strategies can be either physical or chemical, which impacts the physicochemical properties of microparticles, such as their size, morphology, mechanical properties, and degradability.^[^ ^39^ ^]^

**Table 2:** Summary of techniques used for the fabrication of hydrogel building blocks.

Method	Advantages	Disadvantages	Biopolymers	Conjugation	Crosslinking method	Size range (µm)	Reference.
Batch emulsion	High production rate	Large size variation	HA	Norbornene/LAP	Photopolymerization	10‐200	[49]
	Simple and cost‐effective	Removal of organic solvents and surfactant	2‐acrylamido‐2‐methylpropane sulfonic acid	–	Photopolymerization	40, 120	[34]
			PEG	Norbornene/LAP	Photopolymerization	∼200	[59]
			PEG	Azide and alkyne	Inverse suspension polymerization	30–350	[60]
			PEG	Thiol + acrylate	Michael addition	10–40	[55]
			Gel + chitosan	Methacrylate	Photopolymerization	50–310	[61]
			Chitosan	–	Glutaraldehyde	12–64	[47]
			Poly (γ‐glutamic acid)	Hydrazide	Imine bonds	15–30	[62]
			Chitosan	Propylene oxide	Imine bonds	6–16	[62]
			𝜅‐carrageenan	–	Cooling	133	[54]
			Alg	Tyramine	Photopolymerization	94	[46]
			PEG	Acrylate and thiol	Michael addition	>40	[55]
Microfluidic	Precise control over size and infirmity	Complex optimization process	Alg	–	Internal ionic gelation	25‐30	[63]
	Versatility for various applications	Limited scalability	Gel	Methacrylate	Cooling + UV‐light polymerization	70–120	[64]
			HA	Norbornane	Thiol‐ene reaction	100	[65]
			HA	Methacrylate + tetrazole	Tetrazole‐alkene photoclick chemistry	43	[66]
			HA	Norbornene + adamantane	Thiol‐ene reaction	30–150	[67]
			HA	Methacrylate+ gallol	Photoclick chemical crosslinking	90	[68]
			HA + Gel	O‐nitrobenzene +Methacrylate	Thiol‐ene reaction	50–140	[69]
			PEG	Vinyl sulfone	Photopolymerization	50, 90	[70]
			PEG	Vinyl sulfone	Photopolymerization + imine bond	15–105	[71]
			PEG	Thiol + Norbornene	Michael addition	100	[72]
			Agarose	–	Michael addition	63–82	[51]
			HA	Methacrylate	Thiol‐ene photo click chemistry	45	[73]
			HA	Acrylate	Michael addition	–	[74]
			HA + Gel	Thiol + norbornene	Michael addition	150–250	[75]
			PEGDA	–	Photopolymerization	101	[76]
			Silk fibroin	–	Photopolymerization	100–400	[53]
			Gel and chitosan	Methacrylate	Photopolymerization	50–310	[61]
Spraying	Biocompatible	Batch‐to‐batch variations	Alg	Oxidized + methacrylate	Ionic gelation + photopolymerization	>300	[77]
	High production rate		PEG	Norbornene	Thiol‐ene reaction (UV)	200	[78]
			PVA	Methacrylate	photopolymerization	50–1500	[57]
Lithography	Various and controlled topographies	Low production rate	HA	Norbornene	Photopolymerization	130 × (520‐1300)	[79]
Fragmentation	High production rate	Large size variation	β‐cyclodextrin+dextran	Methacrylate	Photopolymerization	32	[52]
	Easy and cost‐effective		HA	Thiol	Disulfide bonds	71–189	[80]
			Alg	–	Ionic crosslinking	–	[81]
			Gel	Methacrylate	Thermal gelation	43–603	[48]
Wet spinning	High production rate	Large size variation	HA	Methacrylate	Ionic gelation + photopolymerization	∼104	[82]
	Easy and cost‐effective		+ Alg				

**Abbreviations**: HA, Hyaluronic acid; LAP, Lithium phenyl‐2,4,6‐trimethylbenzoylphosphinate; Alg, Alginate; Gel, Gelation; PEGDA, Poly(ethylene glycol) diacrylate; PVA, Poly(vinyl alcohol).

To obtain granular hydrogels, microparticles are packed through interparticles crosslinking, which can be either through particle‐particle interaction via the same crosslinking or by introducing a secondary crosslinking mechanism on the surface of microparticles, or by cell‐microparticle and cell‐cell interactions among the microparticles.^[^ ^40^ ^]^ In all approaches, microparticles need to be packed by various means like centrifugation, vacuum‐driven filtration, or gravity‐assisted sedimentation, extensively summarized elsewhere^[^ ^24^ ^]^ (Figure 1b). In this section, we provide a brief overview of the intra‐ and interparticle crosslinking approaches with a few examples related to granular hydrogel applications (Figure 1c).

### 2.1 Microparticle Fabrication Techniques

There are many reported materials designed for microparticle fabrication via several bottom‐up techniques (e.g., electro‐spraying,^[^ ^35^ ^]^ batch emulsion,^[^ ^34^, ^41^ ^]^ microfluidics,^[^ ^42^ ^]^ and lithography^[^ ^43^ ^]^). Besides, top‐down methods can also generate micro‐scale fragments by breaking down the already crosslinked bulk hydrogels via applying external mechanical force (Figure 1a).^[^ ^8^, ^37^ ^]^

The microparticle fabrication techniques and their characteristics have already been reviewed in detail elsewhere.^[^ ^30^, ^40^, ^44^, ^45^ ^]^ For example, Daly et al. discussed various fabrication techniques to develop and characterize microparticles. The authors described the methods including batch and microfluidic emulsion, lithography, spraying, and mechanical fragmentation, and their attributed properties like mechanics, injectability, and porosity.^[^ ^45^ ^]^ Additionally, Wei et al., have focused on different geometries of microparticle generation using microfluidic devices and highlighted their advantages in biomedical applications over traditional techniques.^[^ ^44^ ^]^ Another recent review in 2024 highlighted microparticle fabrication techniques, with primary focus on their application in musculoskeletal tissue regeneration.^[^ ^30^ ^]^

Here, we have provided a brief fundamental overview of the common strategies and summarized some examples, highlighting their merits and limitations in Table 2. The microparticle fabrication methods are diverse, and each offers different characteristics, such as simplicity, controllability of size and size distribution, sphericity, consistency of batch‐to‐batch preparation, and production speed. Moreover, a wide variety of natural (e.g., alginate (Alg),^[^ ^46^ ^]^ chitosan,^[^ ^47^ ^]^ COL, gelatin,^[^ ^48^ ^]^ hyaluronic acid (HA),^[^ ^49^ ^]^ fibrin,^[^ ^50^ ^]^ agarose,^[^ ^51^ ^]^ dextran,^[^ ^52^ ^]^ silk fibroin,^[^ ^53^ ^]^ and 𝜅‐carrageenan^[^ ^54^ ^]^) and synthetic polymers (PEG,^[^ ^55^ ^]^ poly‐L‐lysine,^[^ ^56^ ^]^ poly(L‐glutamic acid),^[^ ^56^ ^]^ and poly vinyl alcohol),^[^ ^57^ ^]^ monomers (e.g., acrylates or methacrylates),^[^ ^13^ ^]^ and oligomers (e.g., end‐functionalized PEG with a molecular weight between 3 and 20 kDa) can be applied to obtain desired parameters tailored to specific applications. Thus, the choice of fabrication technique and materials depends on the required microparticle specificity, quantity, compatibility with the available sterilization techniques, and their compatibility with cells. By tuning the concentration and ratio of chemicals, the mechanical and morphological characteristics of individual microparticles can be adjusted.^[^ ^45^, ^58^ ^]^

### 2.2 Microparticles Assembly Techniques

Granular hydrogel preparation can be achieved by various assembly strategies including centrifugation,^[^ ^10^ ^]^ simple and vacuum filtration,^[^ ^83^ ^]^ gravity‐assisted sedimentation,^[^ ^33^ ^]^ controlled dehydration/rehydration,^[^ ^32^ ^]^ capillary wicking,^[^ ^33^ ^]^ or direct collection by spatula to form a soft viscoelastic hydrogel (Figure 1b). Details of these strategies have been discussed and reviewed by Elbert et al.^[^ ^2^ ^]^ Subsequently, microparticle assembly can be achieved through particle−particle interaction via microparticle jamming transition, microparticle annealing by introducing a secondary crosslinking network, and cell−microparticle and cell−cell interactions, which constrain their mobility and influence the mechanical properties of macroscopic construct.

Particle−particle interactions occur through the reaction of functional groups available on the surface of microparticles, which can further be accelerated by introducing a secondary physical or chemical link to anneal them. Moreover, being able to control the intensity of interparticle crosslinking helps to modulate the host response. Thus, under mechanical load, it is essential to maintain the stability and integrity of microparticles, while in other contexts, dispersing microparticles help maximize endogenous cell response, such as blood vessels infiltration.

To form a granular hydrogel, the particle density should be larger than ≈0.60 to enable the transition from liquid to solid‐like.^[^ ^19^ ^]^ Above this threshold, the interparticle interaction increases microparticle jamming. The particle packing density can be controlled by the intensity of the particle packing process, for instance, by increasing the time of filtration or centrifugation, or using strategies to connect adjacent microparticles, including physical and chemical interactions or binders and additives utilization.

#### 2.2.1 Interparticle Friction Through Jamming Transition

Particle jamming transition occurs when sedimented particles packed sufficiently high, and the results in frictional forces that lead to a solid‐like material. Jammed microparticles behave as a Bingham plastic, exhibiting shear thinning properties, low‐yield stress and rapid recovery of their solid‐like state upon removal of high shear stresses.^[^ ^24^, ^84^ ^]^ Jamming state, the point where the movement of internal microgels is restricted by the motion of their neighboring particles, is closely correlated to the particle‐volume fraction (φ) or packing density. For spherical microparticles, when φ exceeds 0.58, they undergo a jamming transition and reach “loose packing state”, and by applying additional mechanical forces, microparticles continue to pack to reach φ ≈ 0.64, known as “random close packing”. Depending on the shape, size distribution and deformability of microparticles, the packing density can be even greater and exceeds 0.64. Stronger interparticle friction can be also achieved by increasing φ in non‐spherical microparticles with heterogenous sizes and large deformability, thus decreasing porosity.

Interestingly, various jamming techniques (e.g., centrifugation, vacuum‐assisted filtration, sedimentation) result in a wide range of pore spaces. For example, vacuum‐assisted packed NorHA particles fabricated by batch emulsion (spherical shape) and mechanical fragmentation (polygonal shape) resulted in pore spaces of ≈3% and ≈8%, respectively.^[^ ^49^ ^]^ Whereas other studies using spherical microparticles of similar sizes achieved higher pore spaces in the range of 20–40%.^[^ ^60^ ^]^ Furthermore, the jamming behavior of particles is also related to the polymer interactions on the particle surface and influenced by several parameters like polymer type and intra‐particle crosslinking.

Particle‐particle crosslinking is the same primary crosslinking chemistry used for intra‐particle crosslinking. The occurrence of click chemistry reactions between two chemical moieties is an example that utilizes a covalent bond, where microparticles form through the reaction between an electrophile and a nucleophile, such as an amine or a thiol and a vinyl group. Subsequently, after packing microparticles, they can interact with each other through the free crosslinking agents.^[^ ^59^ ^]^ In another study, methacrylated gelatin (GelMA) droplets were first gelled in a cold buffer (oil phase) through the thermos‐reversible gelation of Gel, followed by partially photopolymerization of methacrylate groups to form intra‐particle bonds. Subsequently, when particles are packed together, the residual free methacrylate groups on the surface of microparticles photocrosslinked again to form interparticle bonds.^[^ ^85^, ^86^ ^]^ The limited availability and mobility of chemical moieties at the surface of microparticles hinders the formation of a sufficient number of interparticle bonds. As a result, the interparticle friction approach leads to lower mechanical strength due to the limited number of reactive groups on the microparticle surface, and they might find application for soft tissue engineering and in vitro cell models, where stress‐yielding behavior is similar to those in biological tissues.^[^ ^87^ ^]^ Furthermore, this strategy requires very high control over ratios of reagents and degree of intra‐ and interparticle crosslinking.

#### 2.2.2 Microparticle Annealing

Secondary crosslinking between adjacent microparticles can be used to anneal microparticles together to form a microporous annealed particle (MAP) to enhance mechanical stability and avoid structural disintegration.^[^ ^19^ ^]^ Under mechanical loads of myocardium and skeletal muscle, microparticles might disperse and disintegrate after injection. Furthermore, introducing an additional crosslinking among microparticles can modulate the infiltration rate of endogenous tissue and blood vessels. Interparticle annealing can be achieved either by introducing secondary crosslinking groups that are incorporated into microparticles or additional crosslinking groups that are not consumed during the initial intra‐particle crosslinking process. Afterwards, the presence of excess crosslinking groups can be triggered using an orthogonal stimulus. Covalent and non‐covalent crosslinks can be used to modulate the strength and stability of granular hydrogels. Covalent crosslinking methods are known for their excellent stability and high elasticity through decreasing the particle movement under shear forces.^[^ ^59^, ^64^ ^]^ However, due to rigidity and non‐adaptibility of covalent bonds, they usually fail to remodel the highly dynamic extracellular environment efficiently. Moreover, they often rely on the addition of external stimuli such as light and enzyme, which might compromise cell biocompatibility and complicate their processing as injectable materials. Dynamic covalent bonds have been proposed to improve biological functions by providing higher stress‐relaxation rate of the matrix.^[^ ^88^ ^]^ For example, Muir et al. fabricated an adhesive granular hydrogel based on Schiff‐based interparticle crosslink by combining photocrosslinked norbornene‐modified HA (NorHA) microparticles, which were additionally modified with either hydrazide (Hyd‐NorHA) or aldehyde (Ald‐NorHA).^[^ ^8^ ^]^ The formation of dynamic hydrazone bonds after mixing Hyd‐NorHA and Ald‐NorHA in a 50/50 ratio exhibited a threefold increase in compressive modulus (≈9 kPa), compared to non‐adhesive NorHA control. Moreover, when loaded to 20% strain, the adhesive granular hydrogel maintained its structural integrity after multiple cycles, while the non‐adhesive control collapsed after 1 cycle.^[^ ^8^ ^]^ Non‐covalent dynamic crosslinks, such as ionic interactions,^[^ ^61^ ^]^ metal‐ligand coordination,^[^ ^68^ ^]^ supramolecular assembly,^[^ ^89^ ^]^ and guest‐host reactions^[^ ^66^ ^]^ have been used to enhance microparticles interaction. Hsu et al. designed a shear‐thinning and self‐healing granular hydrogel based on reversible polyelectrostatic interaction of GelMA and methacrylated chitosan microparticles without introducing an external trigger, resulting in a dynamic network with strong cohesive properties.^[^ ^61^ ^]^ The authors observed significant improvement of storage modulus from 100 to 3200 Pa upon mixing positively and negatively charged microparticles, demonstrating that this effect can be attributed to the formation of adaptable electrostatic forces, where various ratios of mixed microparticles can lead to various ranges of moduli.^[^ ^61^ ^]^ Such particle‐particle interactions regulate the extrudability of granular hydrogels, and they can be maximized by increasing microparticle packing density.^[^ ^65^ ^]^ The utilization of densely packed microparticles or the addition of an interstitial matrix with the motive of attaining higher mechanics and better extrudability often lead to lower interconnected microporosity. In order to circumvent this challenge, Ataei et al. developed a nanoengineered granular hydrogel by using 3% w/v nanoplatelets as silicate nanoparticles to attach to the surface of GelMA microparticles to improve interparticle interactions by introducing electrostatic interaction between microparticles, without the need to densely pack microparticles for improving shape fidelity and shear‐yielding behavior.^[^ ^90^ ^]^

#### 2.2.3 Cell−Cell Interactions

Interparticle crosslinking of microparticles can also be achieved by either seeding cells on the surface of microparticles or within the cell‐laden microparticles and allowing them to migrate and form cell−cell connectivity and finally generate a 3D stable shape. This approach eliminates the use of chemicals or external stimuli such as UV that might increase the potential risk of cell toxicity. Matsunaga et al. injected COL microparticles into a mold and discovered that cell‐cell interaction was triggered by seeding cells over their surface.^[^ ^91^ ^]^ Similarly, cell‐coated chitosan microparticles were incubated for 7 days and showed the formation of interparticle crosslinking through cellular bridges and small cell‐microparticle complexes.^[^ ^92^ ^]^ In the context of granular hydrogels, Feng et al. encapsulated bone mesenchymal stem cells in gelatin/HA microparticles and observed that after 14 days of culture in a PDMS mold, microparticles self‐assembled and formed the mold shape, without addition of any crosslinker.^[^ ^75^ ^]^ The Michael addition reaction between vinyl sulfone groups of HA and thiol groups of gelatin were designed to be stoichiometrically equal and sufficient for a rapid reaction, yet insufficient to trigger interparticle crosslinking among microparticles by possible residual functional groups.^[^ ^75^ ^]^ In this approach, the microparticle packing density can be determined by the proliferation rate through adjusting cell seeding density and the amount of cell adhesion sites on the microparticle surface. In contrast, limiting cell−microparticle interactions by changing both the cell's physical and biological environment, through reduced packing density and the absence of cell‐adhesive ligands or materials, thus supporting cell−cell interactions and cellular structures. For example, it has been shown that un‐crosslinked PEG microparticles support cellular self‐organization and formation of multicellular structures. In the absence of inter‐particle crosslinking, fully jammed microparticles were added to coculture of Human Umbilical Vein Endothelial Cells (HUVECs) and fibroblasts (at a ratio of 5:1), with the dilution ratios of granular hydrogel volume‐to‐cell medium volume of 1:0, 1:0.75 and 1:1 to increase the pore spacing between the microparticles. Over 4 days culturing cells among microparticles in a polydimethylsiloxane (PDMS) mold, extensive cellularity and network formation were observed by reducing packing density with either 1:1 or 1:0.75 dilution ratios. Furthermore, the influence of cell−cell and cell−material interactions on cellular self‐organization were assessed by adding arginylglycylaspartic acid (RGD) ligands. The cytoskeleton staining network revealed that in the absence of RGD, higher cellularity (twofold increase) was observed.^[^ ^55^ ^]^ The main disadvantage of such an assembly might be the waiting time required for cell proliferation and the whole assembly process. Besides, with the increase of cell number and ECM production, the pores might block and hinder the exchange of oxygen and nutrients.

## 3 Understanding the Structure‐Property Relationship in Granular Constructs

Currently, most hydrogels fabricated using top‐down approaches lack biomimicry properties. Unlike many natural tissues that consist of spatially ordered 3D cellular modules or microstructures (e.g., islets in the pancreas, lobules in the liver, and nephrons in the kidney),^[^ ^93^ ^]^ top‐down fabrication techniques often offer low controllability over precise assembly of building blocks.^[^ ^94^ ^]^ In comparison, researchers are exploring bottom‐up approaches, composed of 3D building blocks that can better resemble the structure of natural tissues by providing accurate positioning of multi‐type cells and designing programmable tissue‐specific models with desired heterogenicity and cell densities.^[^ ^40^, ^45^ ^]^ As shown earlier in Table 1, two main factors that have made the concept of granular hydrogels a significant paradigm shift in biomaterials during the last decade are the inherent microscale porosity within the interstitial voids between microparticles that influences the biophysical, biochemical, and biological properties, and the extrudability with higher polymers concentrations.

Besides, the degradability of granular hydrogels can allow for further interconnected microporosity in the system. As granular hydrogel degradation is mostly dependent on the microparticle degradability rates, it can be tuned by introducing sacrificial microparticles, such as gelatin‐based microparticles, sugar microparticles, or by introducing hydrolytically degradable microparticles.^[^ ^95^, ^96^ ^]^ The decoupling of porosity and biodegradability allows for higher availability of biochemical cues and functional groups introduced on the surface of microparticles. However, there is a compromise between degradability and structural integrity; degradable microparticles can be applied for enhanced cell invasion and tissue remodeling, whereas non‐degradable microparticles maintain the integrity for long‐term cell culture.

In the case of cell‐loaded granular hydrogels, the adaptive microporous structure led to enhanced cell−cell contact, cell migration, cell differentiation, ECM deposition, and vessel infiltration.^[^ ^67^, ^97^, ^98^ ^]^ In the case of cell‐free hydrogel for in vivo tissue engineering purposes, the infiltration of endogenous immune cells, vessel ingrowth, and integration of host tissue are supported. In the present section, we critically delve into the relationship between the internal structure and the physicochemical properties of granular hydrogels.

### 3.1 Microscale Porosity

**Figure 3:**
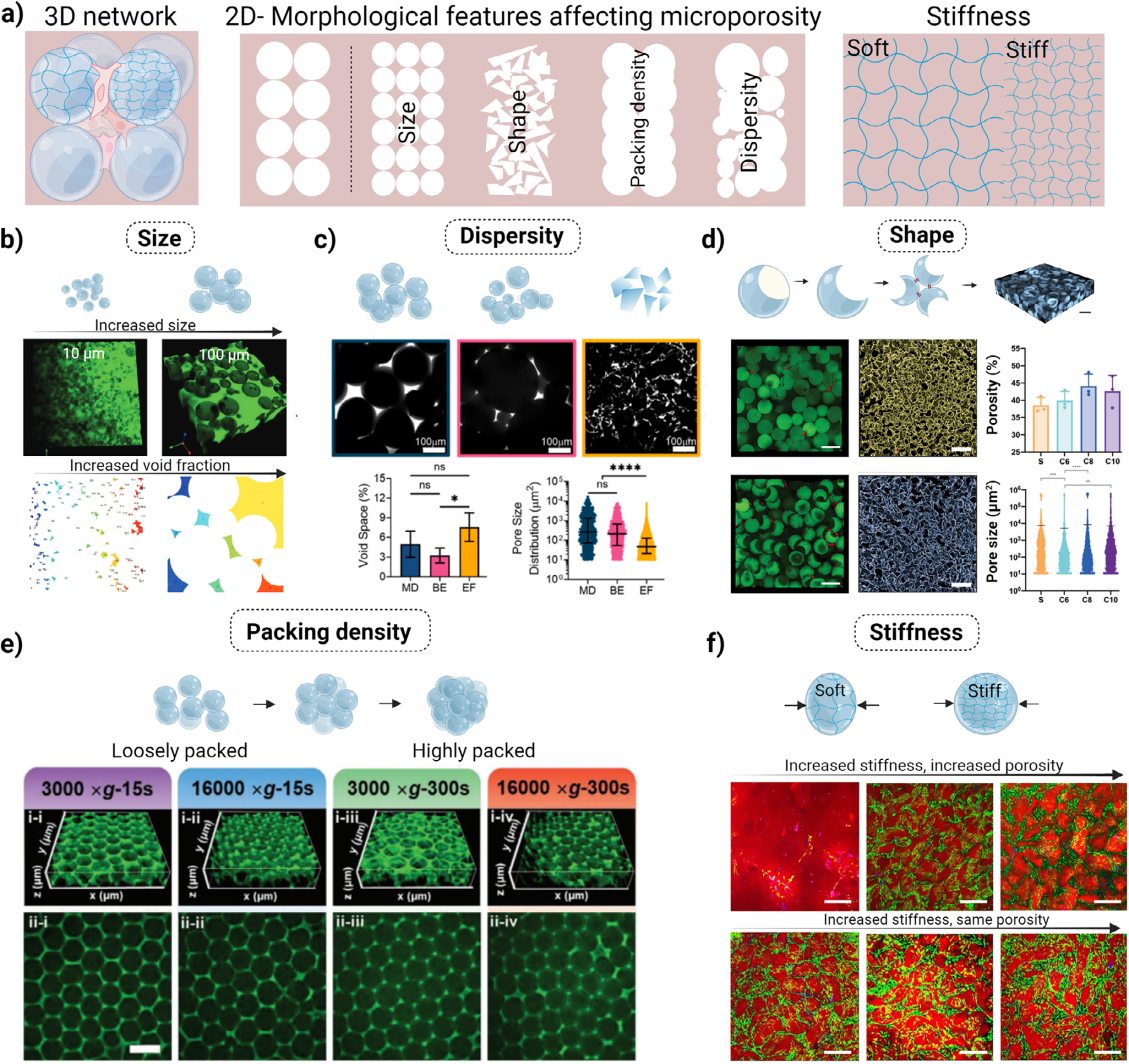
Schematic representing the matrix of granular hydrogel and the effect of various structural characteristics on porosity characteristics. a) 3D and 2D images of granular matrixes with micron‐sized pores. The 2D images show that the size of the mesh is proportional to the particles’ microstructure such as their size, size distribution, morphology, packing density, and stiffness. b) Size: Representative confocal images of 3D granular hydrogel demonstrating the effect of different particles sizes on void fraction. Reproduced with permission.^[^ ^60^ ^]^ Copyright, 2017 WILEY‐VCH GmbH (c) Dispersity: Representative images of norbornene‐HA microparticles fabricated by microfluidics device (MD), batch emulsion (BE), and extrusion fragmentation (EF) and porosity measurements showing void space and pore size distribution. Reproduced with permission.^[^ ^9^ ^]^ d) Shape: 3D confocal images of the top‐view (scale bar = 100 µm) and the interstitial void spaces (scale bar = 200 µm) of spherical‐based granular hydrogel (top row), and crescent‐based granular hydrogel made with 8% sacrificial gelatin (bottom row). Porosity and pore size distribution quantified by IMARIS and FIJI software for granular hydrogels made with spherical (s), and crescent microparticles made with carrying concentrations of gelatin between 6% (C6), 8% (C8), and 10% (C10).^[^ ^98^ ^]^ e) Packing density: Fluorescence images showing void spaces at varying packing conditions (scale bar = 100 µm).^[^ ^17^ ^]^ f) Stiffness: Representative fluorescence images of granular hydrogels prepared by mechanical fragmentation of a photocrosslinked bulk hydrogel. The top images are made with different crosslinking degrees with constant wt.% of microparticles to create a scaffold with varied stiffness and varied porosity, while the bottom images represent scaffolds with varied stiffness but with the same porosity. The spatial relationship between living cells (green) and microparticles (red) after 21 days of incubation shows the dominant role of porosity over stiffness on cell spreading (scale bar = 200 µm).^[^ ^99^ ^]^

As mentioned previously, microscale porosity is the major advantage of granular hydrogel that needs to be rationally designed toward a target application. The ability to modulate porosity in granular hydrogels influences both printability (injectability) and cellular functionality. Further optimization of void fraction could be used to satisfy printability requirements of granular inks. Rearrangement of the void space could be leveraged to a tunable mechanical property required for cellular response. This could also enhance oxygen and nutrient transport throughout the hydrogel, necessary for cell growth and proliferation. If the packing density is low, it can lead to a higher degree of porosity and the rheological properties of the hydrogel might not be sufficient to keep the printed construct stable. Conversely, if the packing density is high, the resultant lower porosity hinders its application for cellular functionality. Therefore, it is important to calculate the porosity before their application for tissue engineering applications. For example, reagents (e.g., high molecular weight fluorescein isothiocyanate–dextran or tetramethylrhodamine‐dextran), microscopes (e.g., light and confocal), and image‐processing softwares (e.g., MATLAB, FIJI, IMARIS) are commonly used to quantitatively and qualitatively assess the porosity;^[^ ^11^ ^]^ however, one limitation of such an approach is that it introduces subjectivity, which yields relative values and hinders comparison across different users. Key structural features, such as microparticle size, interparticle crosslinking degree, and packing densities (**Figure** **3**a), significantly impact pore size distribution, interconnectivity, mechanical properties, and diffusivity; thus all of which play a key role in regulating cellular behavior, nutrient transport, and overall tissue integration.^[^ ^12^ ^]^

#### 3.1.1 The Effects of Microparticle Size and Size Distribution on Microscale Porosity

For instance, Caldwell et al. prepared different sizes of PEG microparticles through inverse suspension polymerization by exposing them to either vortexing (low shear) or sonication (high shear).^[^ ^60^ ^]^ The authors showed that by altering the particle size from 10 to 100 µm, the void fraction ranged between 0.12 and 0.29 (Figure 3b).^[^ ^60^ ^]^ Apart from microparticle size, polydispersity can also influence void fraction, with previous studies showing that using polydisperse microparticles causes the small ones to fill the free voids between larger neighboring microparticles.^[^ ^9^ ^]^ To understand the influence of size distribution on porosity characterization, Muir et al. formulated HA‐based microparticles through three different methods, including microfluidic device, batch emulsion and mechanical fragmentation by extrusion.^[^ ^9^ ^]^ With a similar particle size (≈ 100 µm), microfluidics resulted in spherical homogeneous microparticles, whereas the other two methods resulted in heterogeneous size distribution with a coefficient variation of 30–40% and 25–50%, respectively (Figure 3c). The results demonstrated that the lowest void space of ≈3% was related to microfluidic method, and the highest void space of ∼8% was related to fragmentation method.^[^ ^9^ ^]^

#### 3.1.2 The Effects of Microparticle Shape on Microscale Porosity

Depending on the microparticle preparation methods, bottom‐up or top‐down, their shape can be either spherical or non‐spherical, like microrods, microstrands, and microribbon, with low and high aspect ratios and median diameters between several hundred nanometers and several hundred micrometers. Various studies have explored the well‐established and newly adapted techniques to fabricate non‐spherical microparticles.^[^ ^36^, ^100^ ^]^ A review in 2023 discussed in detail the fabrication methods of non‐spherical microparticles with low‐ and high‐aspect‐ratios and their application in tissue engineering.^[^ ^12^ ^]^ For example, non‐spherical microparticles with higher aspect ratios have shown not only enhanced cell viability, proliferation, and migration, but also increased formation of new tissue by providing better alignment and cell differentiation.^[^ ^10^, ^100^ ^]^ This effect might be due to the fact that high‐aspect‐ratio microparticles can provide larger void spaces.

Suturin et al. developed rod‐shaped microparticles functionalized with 17–29 wt.% amine groups with a width of 10 µm and varying length from 50 to 200 µm, and resulted in high aspect ratio (from 5 to 20). The microparticles were then jammed together via amine‐epoxy reaction and resulted in porosity in the range from 65% to 90% and mean pore size ranging from 39 to 82 µm.^[^ ^100^ ^]^ However, higher porosity results in loose granular hydrogels and can limit the application for some diseases. Tang et al. prepared crescent‐shaped microparticles through aqueous two‐phase separation of PEG and a sacrificial material like gelatin or dextran to create interstitial voids. Different concentrations of gelatin between 6%, 8%, and 10% led to a varied range of cavity sizes, ranging from 68% to 84%, and correspondingly the porosity. Interestingly, 8% gelatin showed the highest porosity (≈44%), which could be attributed to the formation of wider deformable openings on microparticles, and highlights the importance of in vitro optimization of microparticles formulations (Figure 3d).^[^ ^98^ ^]^

#### 3.1.3 The Effects of Packing Density on Microscale Porosity

Besides fabrication method, the packing or jamming conditions (e.g., centrifugation or vacuum filtration duration and magnitude) have the ability to tune void fraction and pore characteristics. In an effort, Jaberi et al. fabricated thermal‐crosslinked GelMA microparticles by microfluidics with diameter of ≈100 µm and relative monodispersity, and assembled them using centrifugation with varying forces (3000 × g or 16 000 × g) and durations (15 s or 300 s), followed by photocrosslinking by exposing them to UV light.^[^ ^17^ ^]^ The authors found that the pores were interconnected independently of packing conditions, however, as the centrifugation force and duration increased, the void fraction decreased. Pore size analysis of fluorescence images showed that the void fraction decreased from 0.19 at the loosely packed condition (3000 × g and 15 s) to 0.07 at the highly packed conditions (16 000 × g and 300 s) (Figure 3e).^[^ ^17^ ^]^

Apart from the packing step required for granular hydrogel preparation, the occurrence of jamming within the nozzle during hydrogel/bioink injection can alter the porosity characteristics. For example, Xin et al. found that the nozzle geometry (size and shape) and syringe diameter affect the packing density, deformability, and the resulting microporosity of granular hydrogels. as they are attributed to the varying shear stresses during the dissipation process.^[^ ^72^ ^]^ The authors demonstrated that the flow initiates only when microparticles are sufficiently packed to create forces that overcome the resistance at the nozzle wall, this further packing arrangement affects how the material yields, especially when extruded through narrow nozzles.^[^ ^72^ ^]^ Potential adhesion and the creation of secondary crosslinking between microparticles as a result of high nozzle shear might also decrease the porosity. Therefore, the nozzle's shape and size must be carefully selected to ensure optimal extrudability while achieving the desired porosity and shape fidelity.

#### 3.1.4 The Effects of Microparticle Stiffness on Microscale Porosity

Notably, the stiffness and the subsequent deformability of microparticles under compression force can also alter the packing density and the porosity. If microparticles are rigid and spherical, the granular hydrogel would show an increased level of porosity and pore size. In contrast, if microparticles are soft and deformable, they can be easily compressed during the packing process and the interstitial space might collapse and result in a much lower porosity. It is worth mentioning that flat‐faced microparticles with perfectly aligned stacking may have a near‐absence of porous network.^[^ ^101^ ^]^ Asadikorayem et al. found that the porosity of granular hydrogels can be modulated by microparticle stiffness through tuning the degree of crosslinker concentration, without the need to change the microparticle size. A zwitterionic (ZI) bulk hydrogel was created by the photopolymerization of zwitterionic monomers in the presence of tetra (ethylene glycol) diacrylate or GelMA, which were then mechanically fragmented to form microparticles, and crosslinked together enzymatically using tyramine acrylamide. The authors indicated that by controlling the stiffness of the microparticle (1–3 kPa), the porosity can be tuned across a wide range of 5–40% (Figure 3f).^[^ ^99^ ^]^ To increase the void fraction volume by other methods than packing density manipulation or sacrificial microparticle incorporation, Segura's group adapted the formation of cryogels to enhance porosity of individual microparticles.^[^ ^102^ ^]^ Microparticles were incubated in a buffer solution containing 200 mm TrisHCL, 150 mm NaCl, 20 mm CaCl_2_ overnight, frozen in liquid nitrogen to form ice crystals, and lyophilized to allow sublimation, and forming an interconnected microporous structure within microparticles, with void volume fraction of 0.4407 compared to the original microparticles having void fraction of 0.287.^[^ ^102^ ^]^

### 3.2 Modularity and Multifunctionality

**Figure 4:**
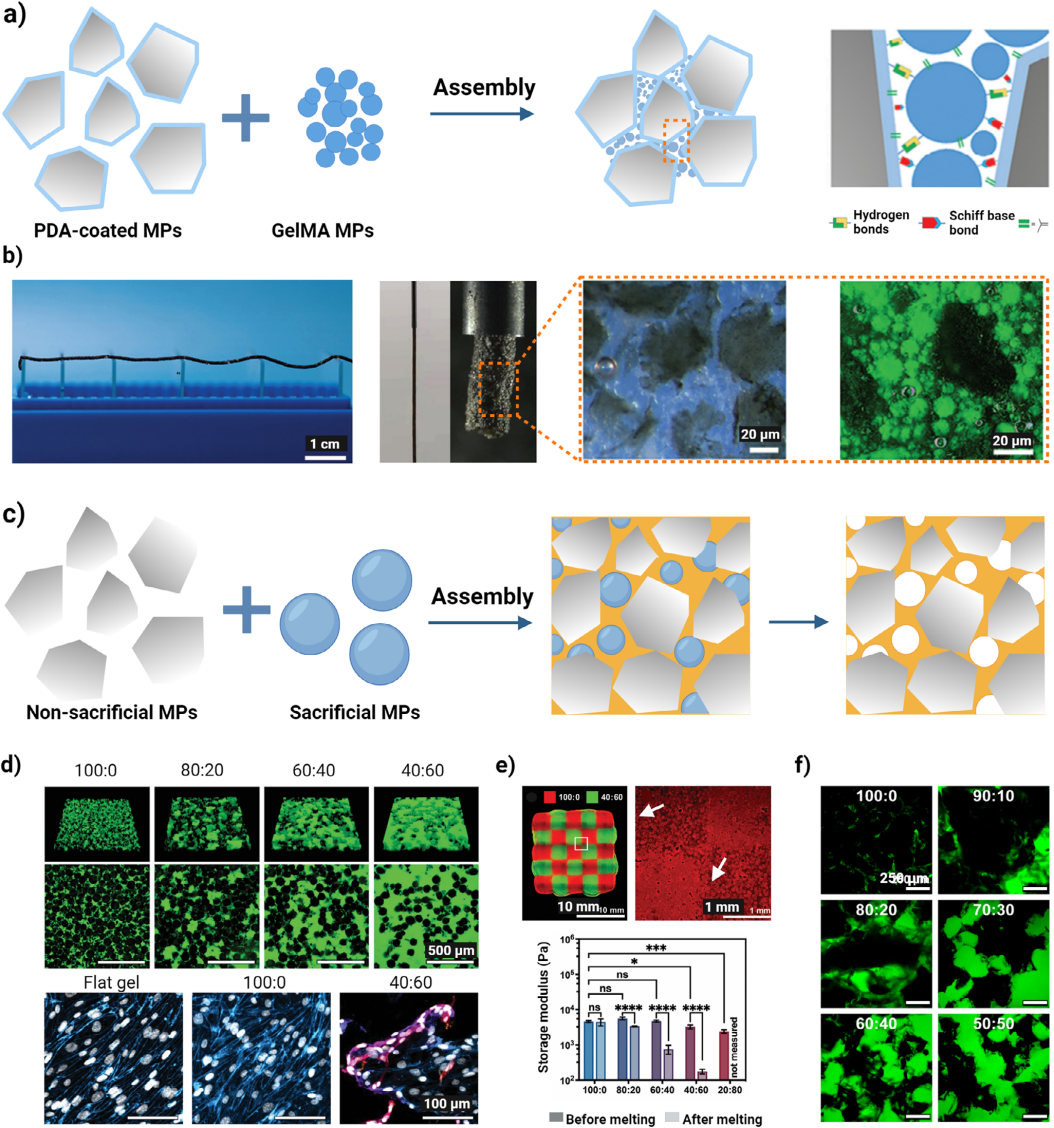
Designing modular granular hydrogels. a) Schematic representing the design of a lubricant granular hydrogel by using GelMA microspheres to act as both crosslinkers for PDA‐coated dECM microparticles assembly and a roller to promote the movement of dECM microparticles. Reproduced with permission.^[^ ^105^ ^]^ Copyright 2023, Wiley‐VCH GmbH b) Representative images of fidelity and stability of extruded filament along with fluorescence microscopy images of the scaffold. Scale bar = 20 µm. Reproduced with permission.^[^ ^105^ ^]^ Copyright 2023, Wiley‐VCH GmbH c) The addition of sacrificial microparticles to non‐sacrificial microparticles to alter the void fraction; d) Representative images of a granular bioink with gelatin and GelMA after UV‐crosslinking and melting to remove gelatin. The ratio of GelMA: gelatin affects void fraction (microparticles are in black and voids are in green), and cell infiltration at day 7. Scale bars = 500 and 100 µm; e) A printed scaffold of two different composites, and subsequently after photocrosslinking and gelatin leaching, shows the diffusion of Rhodamine B (red) into the void structure (regions defined with white arrows). Scale bars = 10 and 1 mm, and storage modulus of pre‐ and post‐melting with different microparticle ratios. Reproduced with permission.^[^ ^106^ ^]^ Copyright 2021, Wiley‐VCH GmbH. Statistical significance was tested by one‐way ANOVA with Tukey's post‐hoc analysis; *p < 0.05, **p < 0.01, ***p < 0.001, ****p < 0.0001. f) Void fractions within 3D printed GelMA granular scaffold containing oxidized Alg as sacrificial microparticles with various GelMA:Alg ratios of. Scale bars 250 = µm. Reproduced with permission.^[^ ^48^ ^]^ Copyright 2023, Royal Society of Chemistry.

The possibility of mixing multiple microparticles with various physicochemical (e.g., different compositions, shapes, and sizes) and biological properties (e.g., loading diverse cells and drugs) within a single hydrogel may be advantageous for mimicking the complex mechanisms of tissue regeneration.^[^ ^103^, ^104^ ^]^ By modulating the dynamic interaction between microparticles, it is possible to achieve a granular hydrogel with sufficient stability, extrudability, and moldability tailored to the desired application. Zhang et al. developed a heterogeneous ECM‐based granular hydrogel, inspired by a roller to reduce the rolling friction when moving heavy objectives (Figure **4**a).^[^ ^105^ ^]^ The authors introduced spherical GelMA microparticles with mass content of 60–70 wt.% into friction pairs of decellularized extracellular matrix (dECM) microparticles (40–30 wt.%) through the void space to act as a crosslinker for PDA‐coated dECM microparticle movements, such as friction, squeezing, deformation, and collision. The printability results revealed well‐performed extrudability and fidelity of the “lubricant granular bioink” (Figure 4b).

Another study incorporated radiopaque zirconium oxide (ZrO_2_) nanoparticles into fragmented microparticles (ranged in diameter from 10–1000 µm) for generating radiopaque granular hydrogels for imaging purposes.^[^ ^107^ ^]^ Norbornene‐HA granular hydrogels with and without 30 wt.% ZrO_2_ could be extruded through gauge needles of 25G without excessive force, and resulted in storage modulus of ≈8 and ≈5 kPa and compressive modulus of ≈35 and ≈20 kPa, respectively. The slightly higher moduli in ZrO_2_ group are likely due to the frictional interactions between microparticles. The resultant radiopaque granular hydrogel could be extruded through displayed shear‐thinning and self‐healing behavior, as well as limited diffusion of ZrO_2_ over a period of at least 4 weeks to retain the radiographic signal within matrix.^[^ ^107^ ^]^ Coagulative granular hydrogel is another category of functional granular hydrogel that has been introduced through the assembly of thrombin‐functionalized GelMA microparticles. Deng et al. found that thrombin‐immobilized GelMA microparticles exhibited 37% catalytic activity (≈0.11 U mg^−1^), compared to the negligible thrombin activity in unfunctionalized and BSA‐functionalized microparticles, and proved to form a densely packed fibrillar hydrogel after 24 h by catalyzing blood plasma fibrinogen, which can act as a biological glue to improve hydrogel stabilization and also provide support for endogenous tissue repair.^[^ ^108^ ^]^ Stimuli‐responsive granular hydrogels can be designed to modulate the properties of the hydrogel by changing the temperature and pH of the solution. Sayed et al. printed thermo‐responsive pNiPAM microparticles incorporated with terpyridine ligand crosslinkers, then treated them with iron (II) ions to form a strong complexation between [Fe^II^Tpy_2_]^2+^ metal‐ligand bonds and terpyridine moieties by increasing temperature above the lower critical solution temperature (LCST). With reversible thermos‐responsive behavior, the granular 3D‐printed construct could undergo swelling/deswelling based on iron oxidation.^[^ ^109^ ^]^ Upon increasing the temperature up to 50 °C, the thickness of strands decreased to 234 µm (among the thinnest reported strand sizes), while it was fully reversed when the temperature decreased back to 20 °C. Moreover, by increasing the pH value to 9, the scaffold was irreversibly disintegrated completely after 2 h.^[^ ^109^ ^]^ Another responsive granular hydrogel was introduced by using dithiolane‐modified gelatin, as a model protein, that can behave as a multi‐responsive and multi‐functional biomaterial.^[^ ^110^ ^]^ Dithiolanes are ring‐strained and can undergo ring opening to form a dynamic disulfide bond in response to heat and light, and thus, have the potential to be replaced by photoinitiated biofabrication. Efforts have been made to regulate the porosity and in vivo degradation by generating a heterogeneous granular hydrogel through engineering the pores of building blocks themselves, for example, by combining non‐sacrificial and sacrificial microparticles (Figure 4c). Seymour et al. tuned the microporosity in acellular printed scaffolds using a mixture of sacrificial gelatin microparticles and non‐sacrificial GelMA microparticles.^[^ ^106^ ^]^ Under a constant polymer content, the void fraction was altered from 0.2 to 0.57 by changing the ratio of gelatin to GelMA microparticles (Figure 4d).

To mimic tissue structures, they also printed a two‐materials chessboard pattern with 100:0 and 40:60 microparticle ratios of GelMA to gelatin, and the scaffold remained intact after melting sacrificial gelatin microparticles, with distinctly different microporosity. In addition, rheological data demonstrated that higher portion of sacrificial gelatin microparticles led to a significantly lower storage modulus (Figure 4e). In their following study, oxidized Alg microparticles were utilized as sacrificial microparticles in combination with GelMA with various ratios and resulted in void fractions from 0.03 to 0.35 (Figure 4f).^[^ ^48^ ^]^ Apart from controlled void fraction, rapid cellularization and depth‐independent cell distribution are also achieved by the encapsulation within sacrificial microparticles.

### 3.3 Shear‐Thinning and Self‐Healing Properties

Granular hydrogels exhibit shear‐thinning and self‐healing properties that facilitate the extrudability through a nozzle that make them suitable for injectable hydrogels and extrusion‐based bioprinting.^[^ ^26^, ^65^ ^]^ Injectability of granular hydrogel allows them to be used as a filling biomaterial for arbitrarily shaped volumetric defects for soft and dense tissues. Granular hydrogels exhibit viscous‐like behavior at higher strains and self‐recovery at lower strains, which relies on physical or chemical interlocking of packed microparticles. The interlocking bonds break when the force is exerted, and the material is back to its initial state when it is removed.^[^ ^111^, ^112^ ^]^ The combination of yield‐stress, shear‐thinning, and self‐healing behavior of granular hydrogels enables their flowability through a nozzle and the integrity of the deposited gelatin without relaxation. Such superior rheological properties of granular hydrogels enable the emergence of a wide range of granular‐based injectable hydrogels and bioinks,^[^ ^26^ ^]^ Various polymers, such as gelatin,^[^ ^113^ ^]^ Alg,^[^ ^46^ ^]^ HA,^[^ ^114^ ^]^ dECM^[^ ^105^ ^]^ and various crosslinking strategies have been successfully used to form a granular bioink. The long‐term stability of granular hydrogels in liquid medium, however, is limited under the weak support of initial interparticle interactions and friction among them.

**Figure 5:**
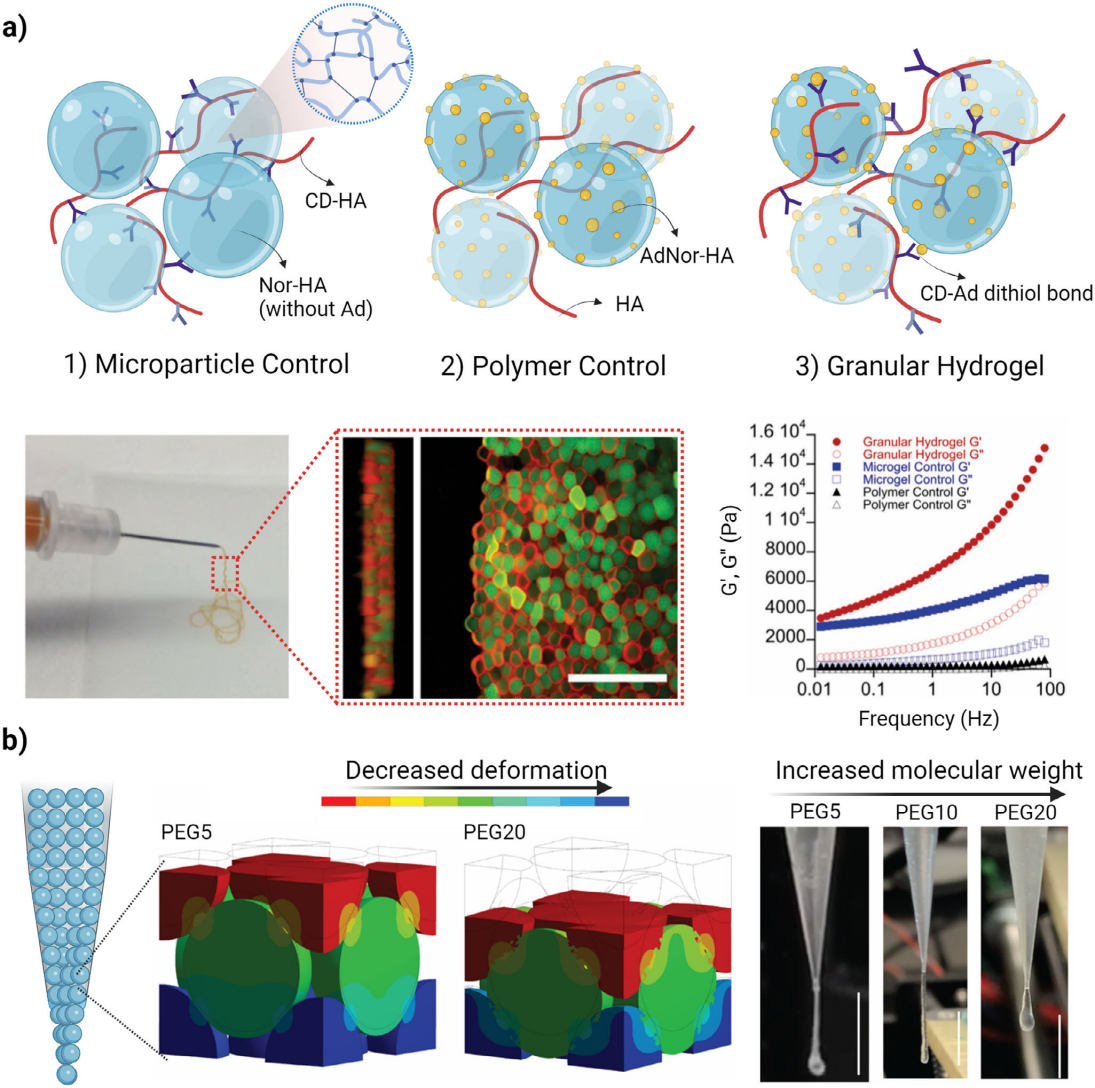
The effect of chemical and physical factors on rheological properties of granular hydrogels. a) The role of interparticle networks on rheological properties. Schematic representation of granular hydrogel formation based on guest‐host interparticle crosslinking. HA microparticles containing adamantane and norbornene (AdNor‐HA) and mixed with cyclodextrin‐modified HA polymer (CD‐HA). The other groups (images 1 and 2) are “microparticle control” and “polymer control”, which are used to investigate the effect of interparticle crosslinking. Ejection of granular hydrogel from a 27G nozzle, and the corresponding confocal image shows the formation of interparticle crosslinking (rhodamine‐labeled CD‐HA and fluorescein‐labeled microparticles). Scale bar = 200 µm. Frequency sweep for three groups, indicating higher modulus of granular hydrogel (red), compared to microparticle (blue) and polymer (black).^[^ ^66^ ^]^ b) The effect of shear stress of the nozzle on rheological properties. Computational simulation showing the effect of PEG‐norbornene molecular weight (PEG5, PEG10, and PEG20) on deformability when gradual force up to 15 µN is applied. Filament extrusion experiment for PEG5, PEG10, and PEG20 shows that the stiffer bioink (PEG5) generated longer hanging filament.^[^ ^72^ ^]^

As discussed earlier, the involvement of a secondary crosslinking among microparticles, mainly through dynamic covalent bonds, can be used to deal with the loss of structural integrity. Mealy et al. used di‐thiol photoinitiation between di‐thiol crosslinker and norbornene as an intra‐particle crosslinking and guest‐host interaction between adamantane and cyclodextrin as an interparticle crosslinking (**Figure** **5**a).^[^ ^66^ ^]^ The authors investigated a series of three formulations, including a “granular hydrogel” group containing 5 wt.% adamantane‐ and norbornene‐modified HA (AdNor‐HA) microparticles, mixed with dithiotritiol (DTT) and 5 wt.% cyclodextrin‐modified HA (CD‐HA) to induce interparticle crosslinking, a “microparticle control” group containing 5 wt.% norbornene‐modified HA (NorHA) microparticles, and “polymer control” group containing 5 wt.% CD‐HA. All hydrogels exhibited solid‐like rheological behavior, characterized by a storage modulus (G′) greater than the loss modulus (G′′), likely due to the dense packing of microparticles. However, “granular hydrogel” group exhibited significantly higher modulus, which is related to the guest‐host interparticle crosslinking.^[^ ^66^ ^]^ Moreover, “microparticle control” group showed higher mechanics when compared to “polymer control” group, probably due to promiscuous interactions among unreacted norbornene and cyclodextrin, as well as hydrophobic interactions. The resulting granular hydrogel also displayed shear‐thinning and self‐healing properties, allowed for injection through a needle size of 27G.^[^ ^66^ ^]^ To improve the performance of a granular bioink for both high resolution and structural stability, the establishment of a post‐printing treatment seems to be necessary. Photo‐mediated free radical crosslinking,^[^ ^115^ ^]^ click reaction,^[^ ^116^ ^]^ and enzymatic crosslinking^[^ ^99^ ^]^ are some examples of post‐printing crosslinking. Surman et al. incorporated Alg methacrylate in ZI precursor solutions with a constitution of 3% of the total solid content, to act as a dual photo‐ and ionic‐crosslinker. After mechanically fragmenting the photocrosslinked bulk hydrogel through 90‐and 50‐µm metal grids, the microparticle inks containing Alg within the structure and on the surface, were immersed in a 100 mm CaCl_2_ bath to induce rapid ionic interparticle interactions, and to make a stable 3D printed construct with ≈20% and ≈15% porosity and 8 and 20 kPa compressive modulus. The authors indicated that the high shape fidelity and resolution obtained is related to the optimum content of Alg that induced a sufficient ionic secondary crosslinking.^[^ ^117^ ^]^

Introducing tunability of the properties can be easily achieved by the combination of different microparticles with multi‐functional properties. In this regard, the effect of microparticle's stiffness and packing density on viscoelastic properties needs to be considered. Hen et al. provided a comprehensive study on the influence of the microparticle's stiffness and packing density on rheological properties of a jammed microparticle suspension, in linear and non‐linear regions. Their results suggest that at low and large strain amplitudes, stiffer microparticles deform less in comparison with soft and medium microparticles. Furthermore, with increasing packing densities, the increase in the yield strain was more pronounced in the soft microparticle beds, at both low and high strains, which was attributed to the larger compressibility/deformability of soft microparticles.^[^ ^118^ ^]^ Deformability of microparticles under compression force can alter not only the packing density and the porosity (as discussed in the section 3.1), but also the rheological behavior when the resistance force from the wall and needle gauge is applied.

One key consideration to avoid significant deformability when force is applied during printing is to improve microparticles’ stiffness. Xin et al. explored the effect of the microparticles’ stiffness on printability performance of the bioink. The authors demonstrated that PEG microparticles with higher stiffness (PEG20) resulted in improved shape fidelity compared to the softer one (PEG5), due to an increased shear stress applied within the nozzle during the extrusion process (Figure 5b).

### 3.4 Mechanical Properties

It has been demonstrated that the compressive modulus of granular hydrogel is typically inferior to the corresponding bulk hydrogels with the same solid content. There are some approaches to overcome this challenge, including modification of the surface of microparticles, for instance, with thiols or metal‐coordinating groups that can bind the adjacent microparticles or by percolating a secondary network.^[^ ^59^, ^64^ ^]^ While introducing microporosity significantly lowers mechanical stiffness relative to bulk hydrogels, Nerger et al. found that tuning porosity can increase viscoelasticity and stress relaxation rate. In this study, a microporous hydrogel was fabricated by a syringe mixing process and achieved a rapid stress relaxation rate when the porosity of the hydrogel reached 30% of the total volume. Microporous hydrogel with liquid‐filled pores relaxed stress at a faster rate compared to hydrogels with air‐filled pores.^[^ ^119^ ^]^

When it comes to 3D bioprinting applications, granular hydrogels can be used as a suspension bath^[^ ^120^, ^121^ ^]^ to provide temporary support for low‐viscous hydrogels or as a bioink with rheological properties, leading to increased mechanical and structural integrity without compromising cellular performance.^[^ ^65^ ^]^ There has been a growing interest in granular bioinks shortly after the introduction of granular hydrogels by Segura's group in 2015.^[^ ^67^ ^]^

In extrusion‐based bioprinting, bioinks must simultaneously meet two opposing requirements; high shape fidelity and cell functionality. Shape fidelity generally depends on high polymer concentrations but restricts proper cellular behaviors like spreading, proliferation, and migration. Granular bioinks have emerged as an innovative approach to decouple viscosity and porosity. Nonetheless, as their stability primarily arises from interparticle crosslinking network, achieving sufficient structural stability in printed constructs remains a challenge.

**Figure 6:**
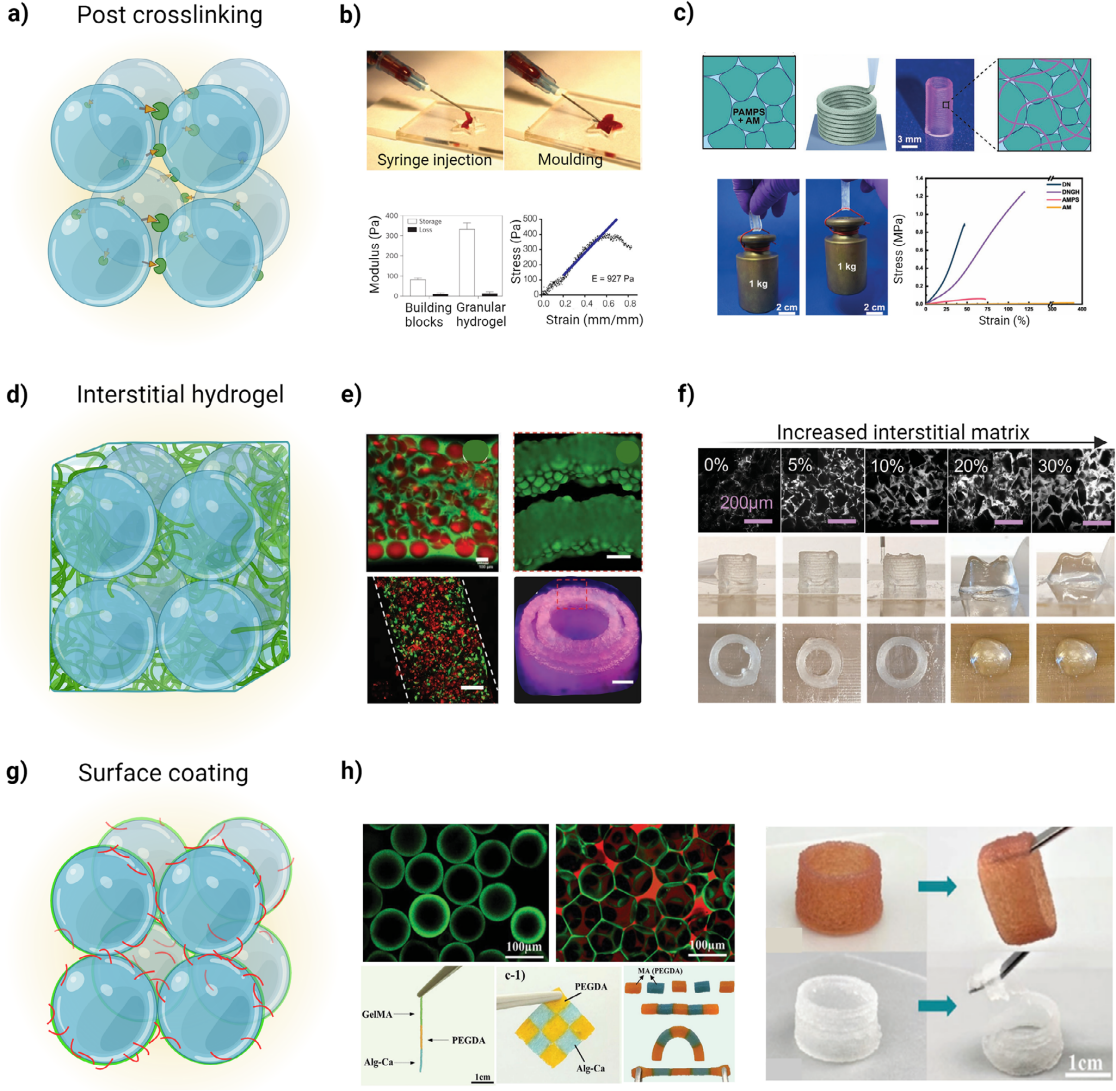
Mechanical reinforcement of granular hydrogels to enhance structural integrity. a) Secondary covalent crosslinking of microparticles through introducing complementary functional groups on microparticles. b) Representative image of microparticle formation using a microfluidic system. Microparticles annealed to one another through FXIIIa to form a granular hydrogel. Images show the injectability and mouldability of microparticles after annealing. Reproduced with permission.^[^ ^67^ ^]^ Copyright 2015, Springer Nature Limited. c) Post crosslinking of 3D printed granular hydrogel through exposure to UV light that can form a percolating network through polymerization. The image of a granular hydrogel stripe (10 × 2 mm^2^) that can tolerate a load of 1 kg weight. Stress‐strain curves display a toughening behavior of double‐crosslinked hydrogel (DN), which is threefold higher than the bulk counterparts (AMPS and AMB) incorporation of a secondary hydrogel precursor solution, known as interfacial hydrogel.^[^ ^34^ ^]^ d) Incorporation of a crosslinked interstitial matrix to improve granular constructs integrity. e) Representative images of microparticles as discrete phase (red) and an interstitial hydrogel (green) as continuous phase. 3D printed fibers with two different cell models (HepG2 in red and HUVECs in green) on day 3 (scale bar = 200 *µ*m). 3D printed double rings crosslinked upon exposure to UV light (405 nm) and its confocal image (microparticles are marked with red and the interstitial hydrogel with green).^[^ ^9^ ^]^ f) 3D printed hollow cylinders with composite granular hydrogel. It shows that by increasing the interstitial matrix percentage from 5 to 30%, the printing success decreases with collapse of structure for >20% of interstitial matrix added.^[^ ^9^ ^]^ g) Surface coating. h) Fluorescence images showing the distribution of the HA‐EGCG (green) as a universal coating additive, and HA‐PBA (red), as a microparticle assembly agent. Spatial heterogenous filament consisted of GelMA, PEGDA and *calcium‐*Alg*, s*elf‐healing of homo‐ and hetero‐granular bioink*, and s*tructural integration of multiple materials after 3D printing by hetero‐bioink. Reproduced with permission.^[^ ^76^ ^]^ Copyright 2024, Wiley‐VCH GmbH.

Therefore, the type of interparticle crosslinking as a post‐printing step, can play a key role on the stability of printed granular structures (**Figure** **6**a).

For example, click chemistry reaction can be utilized to create particle‐particle covalent interactions through mixing microparticles with complementary functional groups of tetrazine‐norbornene and azide‐alkyne pairs. Griffin et al. decorated PEG‐vinyl sulphone (PEG‐VS) microparticles, with an average diameter of 100 µm, with RGD peptides and two transglutaminase peptide substrates (K and Q), so that they annealed to each other through an enzyme‐mediated amide linkage between K and Q peptides. The addition of factor XIII (FXIIIa) (an enzyme responsible for stabilizing blood clots) led to a threefold increase in storage modulus, from nearly 100 to 300 Pa (Figure 6b).^[^ ^67^ ^]^

Hirsch et al. developed a granular hydrogel based on photo‐cured microparticles based on 2‐acrylamido‐2‐methylpropane sulfonic acid (AMPS) at concentration of 4.83 wt.% with enhanced interparticle adhesion. After immersing AMPS drops in acrylamide monomer (AM) with different concentrations, they swelled and were converted to AM‐loaded microparticles upon exposure to UV light. Interparticle annealing was induced by AM polymerization (PAM) post printing via UV light exposure. The results proved that mechanical properties were strongly influenced by AMPS and AM fraction, as first and second crosslinkers. Young's modulus reached values up to 0.57 MPa with 30% AMPS and 20% AM, which was fivefold and threefold higher than individual crosslinked networks, and toughness reached 0.66 MJ m^−3^ with 25% AMPS and 30%AM. Moreover, the stripe lifting test showed its ability to lift a 1 kg weight for at least five loading cycles, which demonstrated its potential for load‐bearing applications (Figure 6c).^[^ ^34^ ^]^

Another approach to improve mechanical properties and hydrogel integrity is introducing a crosslinked interstitial matrix (Figure 6d). For example, Muir et al. investigated the effect of the addition of varied fractions of a crosslinked interstitial matrix on compressive modulus and printing (Figure 6e,f).^[^ ^9^ ^]^ They found that yield stress and printability of composite bioinks were generally inversely proportional to the volume fraction of interstitial solution. For example, the addition of 5%, 10%, 20%, and 30% interstitial matrix decreased storage moduli in 25%, 50%, 75% and 90%. In another example, Feng et al. printed a microparticle‐based biphasic bioink that comprises GelMA microparticles as a discrete phase, and a GelMA precursor solution as a continuous phase to integrate microparticles (Figure 6g).^[^ ^122^ ^]^

The mechanical properties showed much lower elasticity (owing to the displacement of microparticles) and significantly higher fracture strain of biphasic granular bioink compared to the bulk hydrogel. Although the biphasic bioink offered relatively higher mechanical properties, stretch endurance, and better printing capabilities, the micro‐scale porosity compromised when an additional hydrogel precursor occupies the void fractions between microparticles. Recently, Xu et al. proposed microparticle surface coating strategy by using two independent additives to create a dynamic crosslinking among microparticles.^[^ ^76^ ^]^ The authors employed epigallocatechin gallate‐modified hyaluronic acid (HA‐EGCG) as the first additive to building a uniform coating on the particles’ surface regardless of material type before printing, and phenylboronic acid grafted hyaluronic acid (HA‐PBA) as the second additive that can form a dynamic crosslinking via mussel‐inspired chemistry among the coating layers post‐printing. Homogeneous and heterogeneous microparticle assembly bioinks were fabricated based on GelMA, Alg, and PEGDA microparticles, and the results showed that both bioinks exhibited much higher mechanical strength, self‐healing ability, shape fidelity, and tissue adhesiveness in comparison with non‐coated granular bioinks.^[^ ^76^ ^]^ Moreover, the construction of hierarchical structures via printing homo‐ and hetero‐bioinks proved their integration regardless of material variation at spatial and compositional levels (Figure 6h).

## 4 Biological Response of Granular Hydrogels as Cell‐Free and Cell‐Loaded Modular Platforms

**Figure 7:**
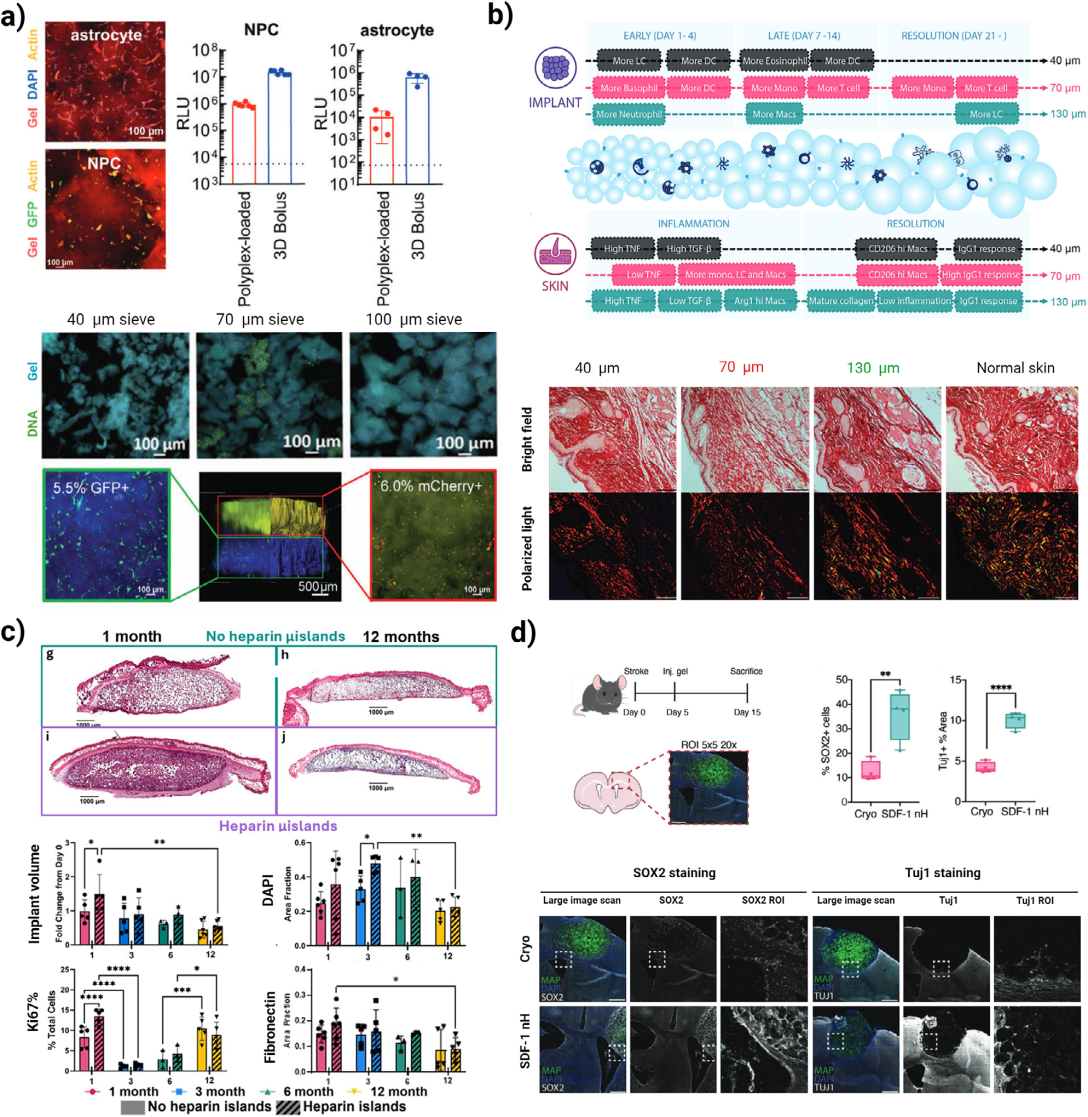
Potential routes of cell‐free granular hydrogels for therapeutic cargo and immunomodulation. a) Confocal images showing the transfection of human astrocytes and mouse‐derived primary neural progenitor cells (NPCs) in 3D culture, using DNA/PEI‐loaded granular hydrogel. Granular hydrogel using fragmented microparticles of different sizes (40, 70, and 100 µm) loaded with DNA/PEI polyplexes, where they show the retention of DNA (green) within the hydrogel (blue, top row), and transfection of two neural cell types in different gel layers, showing the “domain transfection” capability of hydrogels (bottom row). Reproduced with permission.^[^ ^129^ ^]^ Copyright 2021, Wiley‐VCH GmbH. b) Granular hydrogel using spherical microparticles of different sizes (40, 70, and 130 µm) to explore in vivo innate and adaptive immune response in a subcutaneous implant model and a wound healing model, revealing the impact of void size‐dependent immune cells recruitment (top row). Representative Picro–Sirius Red staining images of skin samples after 21 days, demonstrating higher amounts of COL and structurally resembling native skin in wounds treated with the 130 µm‐granular hydrogel. Scale bar = 100 µm. Reproduced with permission.^[^ ^123^ ^]^ Copyright 2023, Wiley‐VCH GmbH. c) Representative images of H&E staining of granular hydrogel with and without heparin µislands, showing their stability and integration after 12‐month subcutaneous implantation in a non‐inflammatory mouse model. Scale bar = 1 mm. Quantification of implant volume, cell infiltration (DAPI staining), fibroblast proliferation (Ki67 nuclear expression staining), and fibronectin (an ECM protein) deposition.^[^ ^130^ ^]^ d) Effect of SDF‐1 NPs delivery on tissue repair for stroked mice. Representative images of sectioned brains stained with SOX2, an NPC marker, and Tuj1, a neuron marker. Both cryo‐based granular hydrogels showed the presence of SOX2+ and Tuj1+ cells after 10 days, while there was a significant increase in the SDF‐1 NPS‐ incorporated hydrogel, which indicates the role of SDF‐1 NPs in accelerated recruitment of Tuj1+ and SOX2+ cells both surrounding and within the stroke. Scale bar = 500 µm. Reproduced with permission.^[^ ^102^ ^]^ Copyright 2023, Wiley‐VCH GmbH.

**Figure 8:**
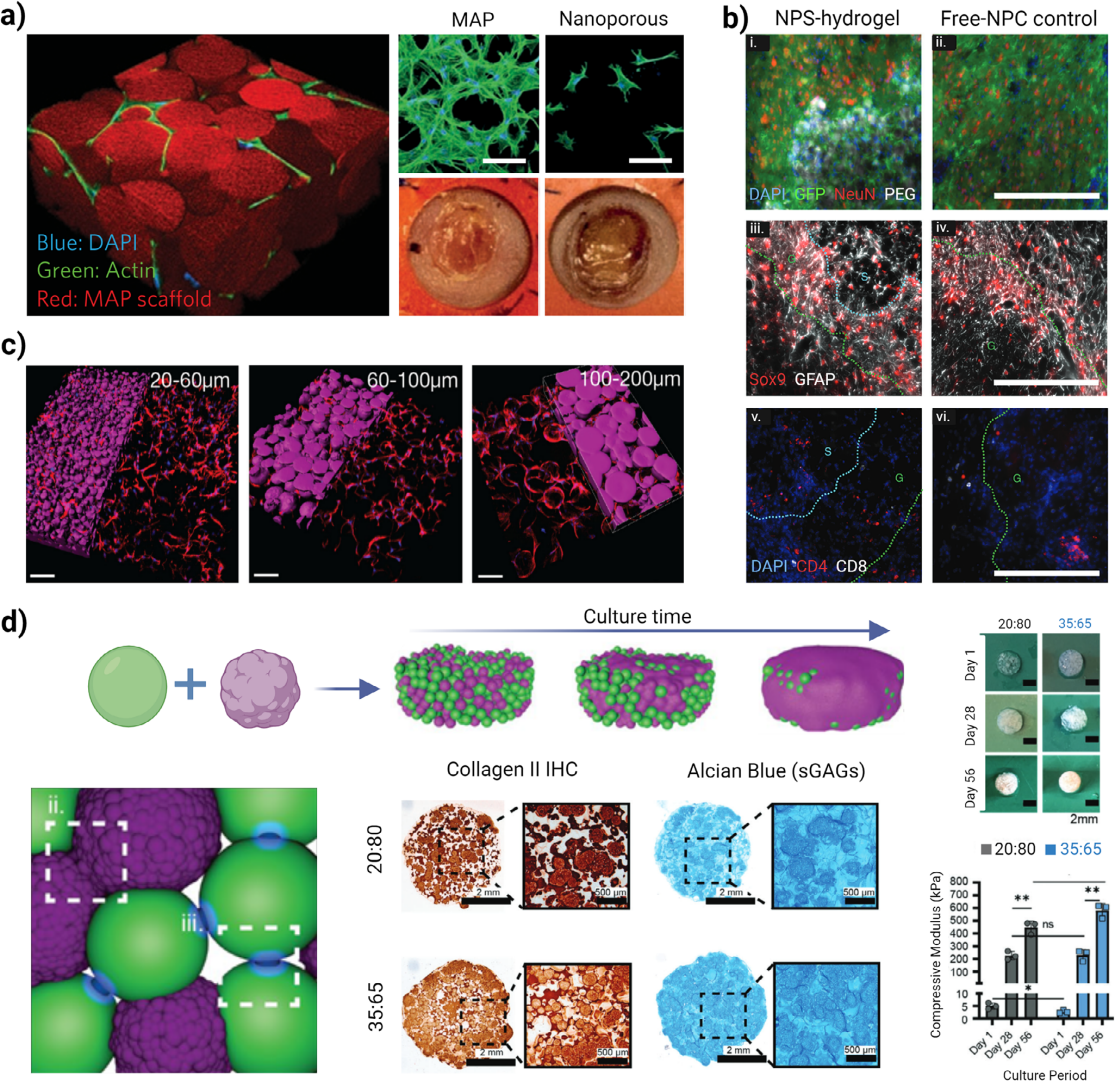
Potential routes of cell‐embedded granular hydrogels as cell carrier or in‐vitro models, and spheroid formation and growth. a) Cells embedded inside microparticles or within the pores among them. Cells encapsulated in PEG‐based granular hydrogel, also termed as microporous annealed particles (MAP), representative fluorescent and wound closure images show significantly higher formation of cellular network in MAP after 6 days and rapid wound closure in vivo, compared to nanoporous hydrogel. Reproduced with permission.^[^ ^67^ ^]^ Copyright 2015, Springer Nature Limited. b) Transplantation of neural progenitor cells (NPC, green) with granular hydrogel (white) showing the ability of cells to differentiate toward mature neurons (NeuN+, red), astrocytes (Sox9+, red) and oligodendrocytes (GFAP+, white), as well as adaptive immunity recruitment (CD4+ in red, and CD8+ in white related to T‐helper cells and cytotoxic T‐cells, respectively.) without significant difference with NPC‐free control group. Scale bars = 200 µm. Reproduced with permission.^[^ ^134^ ^]^ Copyright 2024, The Authors. c) Effect of microparticle diameter on fibroblasts spreading in granular hydrogel after 2 days of culture. Reproduced with permission.^[^ ^49^ ^]^ Copyright 2019, Acta Materialia Inc. Published by Elsevier Ltd. d) Schematic overview of granular composite design, where mesenchymal stem cell (MSC) spheroids and NorHA microparticles are mixed to enable cell–cell contacts over time for where spheroid fusion and growth result in enhanced chondrogenesis and spheroid fusion for tissue formation. Reproduced with permission.^[^ ^124^ ^]^ Copyright 2024, Wiley‐VCH GmbH.

The microporous structure of granular hydrogels makes it possible to create interstitial spaces and voids for infiltration of a wide range of cells and diffusion of biological factors, biomaterial‐cell interactions, angiogenesis, and reduced fibrosis and scar formation.^[^ ^15^, ^123^ ^]^ Thus, by providing intrinsic immune‐instructive properties, granular hydrogels can offer better biomaterial−cell interactions and alteration of immune responses. Not only the size of the pores, but also the shape of the void spaces can create spatial constraints for the infiltrating of cells, thereby influencing cell phenotypes, a pivotal aspect in preventing the appearance of chronic inflammation. Moreover, biological factors like nucleic acids, proteins, peptides, antibodies, small molecule drugs, adjuvants, and ECM components can be introduced to granular hydrogels by conjugating them to the surface of microparticles or by loading them within the matrix.^[^ ^28^ ^]^ The effect of cell spheroids or cell suspension incorporation within the pores on cell outgrowth, cell condensation, and angiogenesis can be also investigated through these platforms.^[^ ^11^, ^124^ ^]^ The tissue repair outcome is dependent not only on the materials of the injected or implanted hydrogel such as their degradability and surface chemistry, but also on the kind of innate immune system response. Here, rather than generally highlighting the application of these materials in various tissues, we focus on four categories of potential routes consisting of therapeutic cargo, immunomodulation, in vitro cell model, and cell spheroid carrier, through which granular hydrogels promote tissue regeneration stimulation (**Figures** **7** and **8**). Additionally, as an updated summary of granular hydrogels’ biological outcomes, we highlighted the most recent in vivo studies for a wide variety of tissue repair applications, such as cardiac, cartilage, wound, muscle, neuronal, foreign body reaction (FBR), disc, and spine, as shown in **Table** **3**. We refer interested readers to excellent reviews focused on tissue regeneration from granular hydrogels and hydrogel microparticles.^[^ ^30^, ^40^, ^45^, ^79^, ^125^ ^]^

**Table 3:** Overview of the in vivo application of granular hydrogels for tissue repair.

Application	Therapeutic/Cell type	In vivo effects of granular hydrogels	Animal model	Reference
Cardiac	RHO labelled dextran (RHO‐DEX) or FITC‐BSA	Unique disease‐dependent behavior; Cleavable material preferred to degrade in the MI condition, while stable microparticle was observed with rare significant differences between normal and MI conditions.	MI rat model	[66]
		High levels of cell invasion.		
	–	Reduced left ventricular remodeling	MI rat model	[101]
		Improved functional outcomes, including reduced left ventricular wall thinning and higher ejection fraction after 6 weeks		
		Locally reduced COL deposition		
	IL‐10	Mitigated inflammation near the injection site after 4 weeks	MI rat model	[137]
		Increased scar thickness after 4 weeks		
		Increase in ejection fraction, cardiac output, dP/dtmax, and preload recruitable stroke work after 4 weeks		
	–	Mitigated remodeling, assessed by improved infarct thickness and reduced left ventricular dilation	Ovine posterolateral model	[138]
		Improved ejection fraction after 8 weeks		
	MSC	The armor prolonged MSCs residence at the MI site	MI rat model	[139]
		Reduced local inflammation and fibrosis		
		Improved cardiac function, including left ventricular wall thickness, left ventricular ejection fraction and left ventricular fraction shortening after 28 days		
Cartilage	Chondrocytes	Enhanced cartilage and subchondral bone regeneration	Rabbit osteochondral defect and wound model mice	[105]
		Higher GAGs and COL II expression		
		Faster wound healing and epithelialization		
		Increased angiogenesis (CD31 expression) and keratinization (CK14 expression)		
	hACs	Small‐diameter microparticles (40 µm) exhibited higher tissue maturation as well as low immunogenicity	Nude mice	[114]
		Larger microparticles (100 and 500 µm) let to increased inflammatory response and lower stability		
Muscle	TSG‐6	Increase in regulatory T cells from day 3 to day 7	Rotator cuff	[140]
		Heparin led to higher macrophage response and level of muscle regeneration markers (eMHC and CLN)	injury rat	
		Sustained delivery of TSG‐6 over 2 weeks	model	
	Acellular	Substantial endogenous cell invasion (12% and 7% depending on the particle size) Infiltration of Pax+ myogenic cells and eMHC+ myofibers compared to 0% cell invasion in bulk hydrogel	VML mouse	[141]
			model	
	RGD	Significant implant retention after 4 weeks compared to nanoporous scaffold	VML mouse	[132]
		No fibrotic capsule formation after 12 weeks	model	
		Higher macrophage polarization toward M2 (CD68+/CD163+) at week 4		
		Moderate level of angiogenesis		
		Innervated myofiber formation as early as 4 weeks, unlike nanoporous structure, but misaligned		
Wound	RGD, HDF, AhMSC, BMhMSC	Facilitated cutaneous tissue regeneration, as proved by the tissue formation within five days	Mice	[67]
		Accelerated wound closure compared to non‐porous controls, complete remaining of granular hydrogel within wound area after 5 days, relative to 60% for non‐porous hydrogel.		
		39% wound closure after 7 days, significantly greater than the no‐treatment control (19%) and the non‐porous control (7%)		
	Zn‐nHAp	Accelerated wound healing process in fused granular hydrogels doped with 10% Zn; as proved by a wound closure rate of ≈96% by day 14	SD rats and Bama	[142]
		Showing strong antibacterial activity against common wound pathogens, such as *Staphylococcus aureus* and *Escherichia coli*	minipigs	
	nH	Enhanced vessel formation and maturation compared to untreated wounds	SKH‐1 mice, C57B6/J mice	[143]
	RGD	Large‐sized (130 µm) microparticle‐scaffolds recruited significantly more neutrophils and exhibited a balanced pro‐regenerative macrophage response	Mice	[123]
		Promoted a timely resolution of inflammation, with lower levels of pro‐inflammatory markers compared to smaller‐sized microparticles		
	RGD	Accelerated degradation and immune infiltration induced formation of adaptive immune response (CD11b+ myeloid cells recruitment) for skin regeneration in D‐peptide granular hydrogel after 25 days	B6 or B6.Rag1^−^/^−^ mice	[97]
	Bioglass particles/siRNA	Accelerated wound closure, angiogenesis, COL deposition and inhibitory function on MMP‐9 expression in the wound region by day 16 compared to untreated group, but akin wound healing ability to bulk hydrogel	Diabetic mice model	[144]
Neuronal	nH and clustered VEGF (hcV)	Increased proliferation and endothelial cell area within the stroke cavity at both 2‐ and 16‐weeks post‐stroke	PT stroke mice model	[145]
		Promoted neurogenesis; proved by increased proliferation rate of neuroblasts (Dcx‐positive cells) in the stroke area		
	HDFs	Reduced inflammatory monocytes (CD11b+ cells)	PT Stoke model of C57BL/6 mice	[146]
		Decreased astrocytic scar thickness		
	SDF‐1α‐loaded heparin‐norbornene nanoparticles	Significant vascularization within the stroke infarct at day 10 post‐injection	Stoke model of C57BL/6 mice	[102]
		Increase perfused vasculature and recruitment of SOX2 NPs		
		Increased neuronal differentiation and vascularization		
		No significant changes in glial scar thickness or loaded in NPs microglial/macrophage infiltration, suggesting that the mechanism of action primarily involves NPC activation and recruitment		
	RGD	Reduction in astrocytic scar thickness after microporous hydrogel injection	Stoke model of C57BL/6 mice	[73]
		Reduction in reactive microglia compared to nanoporous hydrogel		
		Enhanced vascularization in the peri‐infarct area		
	RGD	Reduced cerebral atrophy and preserved NF200 axonal bundles, suggesting a protective effect against long‐term degenerative damage	Mice	[147]
		Increased infiltration of less reactive, pro‐repair astrocytes into the lesion		
		Increased level of reactive astrocytes and microglia, leading to a more inflammatory environment for sham (stroke only) condition		
	Nerve GF	Directs axon outgrowth of up to 4.7 mm in 4 days	Mice	[61]
		Well‐aligned axons with functional recovery after 1 month		
		Degradation of MPs and infiltration of cells after 1 week		
		Neurite outgrowth after 2 weeks		
		Myelin sheathes of regenerated nerve fibers were well‐laminated, close to that of the gold standard at 60 days		
	RGD	Reduced highly reactive astrocytes expressing pERK1/2 in the sham condition (from 56% to 25%) after two days post‐injection of granular hydrogel	Mice	[147]
		Reduction in cerebral atrophy and better preservation of nigrostriatal bundles in mice treated with granular hydrogel		
		Infiltrate of astrocytes to the lesion over time, with significant infiltration observed as early as two days post‐injection and reaching close to 500 µm into the lesion by day 30		
	VEGF NPs	Higher branched sprouting of Endothelial cells in α3/α5β1‐specific matrices, whereas αvβ3‐specific matrices resulted in clustered sprouting and chaotic vascular patterns	Mice	[148]
FBR	RGD/ Bone marrow‐derived macrophages	Similar cell infiltration rate in all sizes of microparticle groups	Mice	[15]
		Increased vascularization in all groups		
		Higher expression of pro‐regenerative marker CD206 in the 70 µm group compared to the other sizes of microparticles		
		Higher amount of macrophage infiltration in the 130 µm group compared to the other sizes of microparticles		
	THP‐1 cells	Porosity‐dependent manner (ranging from 14% to 21%) of host cells (macrophages and mature blood vessels) infiltration;	Mice	[149]
		Significantly higher cell infiltration in HA‐granular hydrogel compared to zwitterionic (ZI)‐granular hydrogel with similar porosities		
		Fibrosis capsule formation around ZI‐granular and bulk implants and HA‐bulk implant, highlighting the dependance of FBR on both materials’ composition and architecture		
Disc	Radiopaque zirconium oxide NPs for imaging	Halted loss of disc height over 8‐week period	Goat	[107]
		Increased proteoglycan and reduced COL contents in the nucleus pulposus region		
		No marked inflammatory response		
Spine	NPC	Astrocyte infiltration into the granular scaffold started at day 14 and support cell differentiation	Mice	[134]

**Abbreviations**: BSA, Bovine serum albumin; MI; Myocardial infarction; IL‐10, Interleukin‐10; MSC, Mesenchymal stem cell; GAGs, Glycosaminoglycan; hACs, human auricular chondrocytes;TSG‐6, Tumor necrosis factor–stimulated gene 6; eMHC, embryonic myosin heavy chain; VML, Volumetric Muscle Loss; RGD, Arginine‐Glycine‐Aspartic; HDM, Human Dermal Fibroblasts; AhMSC, Adipose‐derived human Mesenchymal Stem Cells; BMhMSC, Bone Marrow‐derived human Mesenchymal Stem Cells; GF, Zn‐nHAp, Zinc‐dropped hydroxyapatite nanoparticles; Zn, Zinc; nH, Heparin Nanoparticles; siRNA, small interfering RNA; GF, Growth Factor; VEGF, Vascular Endothelial Growth Factor; PT, Photothrombotic; NPs, Nanoparticles; NPC, Neural progenitor cells; FBR, Foreign body response; THP‐1, human Leukemia Monocytic Cell Line; ZI, Zwitterionic.

### 4.1 Granular Hydrogels as Cell‐Free Platforms for Therapeutics Delivery

Granular hydrogels stand out as a promising entity in the realm of therapeutic cargo, which is primarily attributed to their versatile capabilities in delivering drugs, and bioactive molecules like growth factors (GFs) to provide controllability of biological responses.^[^ ^126^ ^]^ Carvalho et al. utilized a GelMA aerogel‐based granular hydrogel as a non‐viral vector for localized delivery of mRNA lipoplexes (mRNA‐LP) and prolonged transgene expression. High loading efficiency (98%) and homogenous distribution of mRNA within the hydrogel were achieved through the polyelectrostatic interaction between lightly negatively charged polymer and the positively charged mRNA complexes. In vitro tests revealed that fibroblasts could easily infiltrate, attach and proliferate within the mRNA‐LP‐loaded granular hydrogel after 48 h, while 13% of cells were being transfected by mRNA polycomplexes, compared to no observed transfection in the free mRNA‐loaded hydrogel.^[^ ^127^ ^]^

Puiggalí‐Jou et al. fabricated heterogeneous hydrogels based on a mixture of HAMA and 25% GFs‐loaded sulfated HAMA (SHAMA) microparticles with two different sizes of 20 and 150 µm.^[^ ^128^ ^]^ After 6 weeks of explantation on bovine osteochondral, 20 µm‐sized hydrogels showed better cell infiltration and tissue maturation (COL II and GAGs production), likely due to the smaller void space which allowed for higher cell colonization. Moreover, loading GFs in both hydrogel groups resulted in significant stiffness enhancement from initially 10 kPa to 20–100 kPa, which provides evidence for tissue maturation in granular hydrogels, regardless of their size.^[^ ^128^ ^]^

Several studies have investigated the effect of different forms of granular hydrogels for nucleic acid delivery to increase cellular uptake by taking advantage of higher cell infiltration. Granular hydrogels with three different levels of void spaces, ranged from 10–35%, were engineered through fragmentation with sieve sizes of 40, 70, or 100 µm, for embedding DNA/PEI nanocomplexes with high loading capacity for plasmid transfection (Figure 7a).^[^ ^129^ ^]^ To prevent DNA/PEI aggregation inside hydrogel, nanocomplexes were first stabilized through coating with Norbornene‐modified HA at HA/PEI ratio of 5 w/w and 2.5 µg DNA/µL gel. DNA/PEI particles were capable of providing prolonged rates of transfection, over a one‐month period, likely due to the use of metalloproteinase‐cleavable crosslinkers to promote hydrogel degradation. The authors demonstrated that hydrogels successfully transfected human astrocytes and primary mouse neural cell types, which were similar in all hydrogels with various void spaces, suggesting the insignificant role of porosity in fragmented‐based granular hydrogels,^[^ ^129^ ^]^ as opposed to a previous work from the same group with spherical‐based granular hydrogels.^[^ ^49^ ^]^ Aside from exploring the transfection of a homogenous hydrogel, the authors also explored the possibility of “domain transfection”, by creating a two‐layered hydrogel, in which DNA/PEI nanocomplexes in each layer contained only one specific reported transgene. The outcomes showed specific transfection of cells seeded within each particular layer, proving the local release and uptake of DNA/PEI particles.^[^ ^129^ ^]^

Recent findings have shown that the possible relationship between porosity and therapeutic effects is not just related to the pore characteristics, e.g. pore size, pore shape, and pore size distribution, but it also depends on microparticles size. In the context of localized immunomodulator delivery, for instance, Liu et al. explored the in vivo cellular response to various spherical microparticle sizes (40, 70, and 130 µm) with consistent void fraction of 25–35%. It was found that microparticles with a diameter of 40 µm generated microporosity comparable to the diameter of bone marrow‐derived macrophages that reduced the inflammatory response. Instead, microparticles of 70 µm in diameter caused an increase in pro‐reparative Th2 response and pro‐inflammatory reaction by showing an increase in the production of iNOS, CD206, CD86, and MCHII. Microparticles with a diameter of 130 µm showed similar results as 40 µm ones. The difference in microparticle size also affected the morphology of macrophages, which can be related to the M1/M2 polarization: cells tended to stretch and fully occupy the 3D pores in smaller pores (microparticles of 40 µm), whereas cells remained more spherical within larger pores (microparticles of 70 µm). In the least spatially confined group (microparticles of 130 µm) the cells were larger in volume and surface area. Moreover, the assessment of COL architecture and maturity by immunohistology staining showed that 130 µm microparticles promoted mature COL formation in mice, as also proved by the highest average epidermis to dermis ratio (Figure 7b).^[^ ^123^ ^]^

### 4.2 Granular Hydrogels as Cell‐Free Platforms for Immunomodulation

Immune system modulation occurs through cytokine/chemokine signaling, antigen uptake, or receptor interaction essential in vascularization, host tissue integration and innervation.^[^ ^131^ ^]^ Other in vitro and in vivo studies also showed a strong correlation between porosity, pore size, and immune responses.^[^ ^15^, ^123^ ^]^ The limited infiltration capacity in dense mesh networks might prevent the homogenous distribution of loaded biotherapeutic agents and their direct contact with cells, compromising their effectiveness. Moreover, adding microporosity can prolong the therapeutic window of biotherapeutic agents that are embedded in a granular hydrogel and released upon degradation. Thus, this could also decrease the cost of including those bioactive agents in some cases, as a smaller quantity might show similar effect as a larger quantity in a nanoporous hydrogel.

Nicklow et al. studied the effect of long time‐scale subcutaneous implantation of PEG‐based granular hydrogels with and without 10% bioactive heparin µislands (Hep and NoHep) on endogenous cellular response, tissue integration and ECM deposition.^[^ ^130^ ^]^ Both groups maintained their initial volume t 1 month and 45% at 12 months, indicating their stability, and led to extensive cell infiltration even at long timepoints (6 and 12 months) (Figure 7c). Looking at Heparin effect on cellular response, the authors observed that Hep hydrogels led to higher amounts of CD31+ (endothelial marker) cells, CD68, CD4+ and CD3e+ T‐cells infiltration and TE‐7+ (fibroblast marker) cell proliferation at 1 month, with a decreasing trend by 3 months. Like results seen in cellular infiltration, Hep encouraged early ECM deposition by increasing fibronectin, COL I, III and IV markers early on, while resulted in decreased levels at late timepoints, probably associated to the attenuating effect of heparin µislands over time (at 3 and 6 months) and matrix degradation. At 12 months, the increased cell proliferation and decreased ECM deposition could indicate activation of complement system, which caused further inflammation and protease activity (Figure 7c).^[^ ^130^ ^]^

It has been demonstrated that there are three size categories of microparticles capable of modulating the immune response. Small microparticles (1–50 µm) promote the infiltration of single cells and are suitable for the biomaterial‐cell interaction that needs only a low amount of cells such as the growth of small capillaries. Medium microparticles (50–130 µm) promote the infiltration of multiple cells between the interstitial pores, where the orchestration of multicellular scenarios and the cell−cell interaction increases, and the biomaterial‐cell interaction decreases. Large microparticles (>130 µm) promote the infiltration of clusters of cells inside the interstitial pores and again decrease the biomaterial‐cell interactions.^[^ ^7^ ^]^

Ayala et al. investigated the effect of microporosity of a cell‐free granular hydrogel composed of medium microparticles, with diameter of ≈86 µm, on local immune response in a rodent tibialis anterior volumetric muscle loss model. The authors characterized fibrotic capsule formation and found that no fibrotic capsule was observed around the microporous granular implants at 4 and 12 weeks, unlike nanoporous implants that formed capsule with a thickness of 221.7 µm at week 4 and 58.8 µm at week 12. They also showed that CD68+/CD163+ proportion (correlated to M2‐like macrophage phenotype) was more prevalent in granular implants compared to nanoporous implants. As a result of negligible immune response achieved in granular implants, they resulted in significant promotion of angiogenesis and myogenesis, determined by increased CD31+ cell, implant perfusion by 80% and myofiber formation.^[^ ^132^ ^]^

Modulation of physical and chemical properties of biomaterials is also another approach to regulate the biological responses with granular hydrogels. Wilson et al. developed two formulations of microparticles, consisting of highly porous microparticles through cryogel process and microparticles loaded with SDF‐1α heparin NPs, to explore the effect of physical and chemical properties of microparticles individually and when combined, to enhance angiogenesis and endogenous neural progenitor cells (NPCs) recruitment to the stroke region. The results showed higher levels of glucose transporter 1 (Glut1, vasculature marker) in cryo‐microparticles treated mice only 10 days post‐injection, revealing the enhanced vessel infiltration which could be attributed to higher porosity level, despite having no effect on immune cell response within the infarct. The immunofluorescence staining images showed enhancement of Tuj1+ and SOX2+ cells, which indicated NPC recruitment and differentiation were only possible upon SDF‐1 NPs delivery in cryo‐microparticles (Figure 7d).^[^ ^102^ ^]^

Long et al. fabricated discrete HA‐based microrods, which provided biodegradable and bioactive micromechanical signals for reprogramming cells and attenuating cardiac fibrosis.^[^ ^101^ ^]^ The produced HA microrods revealed a range of physiological stiffness and degraded in the presence of hyaluronidase. The in vitro interaction of these microrods with fibroblasts resulted in significant changes in COL expression, proliferation, and other myofibroblast phenotype markers. After introducing into the injured heart, the HA microrods provided anchors to surrounding cells during initial inflammatory processes, attenuating the fibrotic phenotype and improving morphological and functional outcomes in a rat myocardial ischemia‐reperfusion model (MI).

### 4.3 Granular Hydrogels as Cell‐Embedded Platforms as a Cell Carrier or In Vitro Model

Granular hydrogels have been also used in conjunction with cells, whether as a therapeutic carrier of cells or as in vitro models to study the effect of microenvironmental cues on cell behavior. There are two general approaches for spatially locating cells: they can be encapsulated 1) inside microparticles or 2) within the interstitial space among microparticles. For the first approach, cells are mixed with the hydrogel precursor solution, and then the mixture is crosslinked and finally assembled into granular hydrogels.^[^ ^69^ ^]^ Owing to the presence of living cells, the process of microparticle fabrication and assembly should be fast, non‐toxic, and cytocompatible.^[^ ^133^ ^]^ Thus, the choices of materials, crosslinking agents, their concentrations, and gelation and assembly mechanisms become limited. In contrast, the second approach provides a biomimetic 3D environment where cells can rapidly grow and spread, without suffering from the fabrication conditions. Besides, higher cell viability can be achieved due to the facilitated nutrients and oxygen transport, because of interconnected microporosity.

Griffin et al. showed that cells cultured on PEG granular hydrogel exhibited higher proliferation, infiltration, and faster wound healing process compared to bulk nanoporous hydrogel (Figure 8a).^[^ ^67^ ^]^ Tigner et al. fabricated a PEG‐based granular hydrogel, with or without NPCs, to further explore its effect in a mice model of spinal cord injury. In vivo, the cell‐free hydrogels could recruit the host response at days 7, 14, and 28 time points, confirmed by the filled lesioned tissue with β‐IIITubulin, a marker for neurons and axons, and the infiltration of dense population of astrocytes into the hydrogel with upregulated glial fibrillar acidic protein, a marker showing the astrogliosis progression. By loading NPCs within the hydrogels, histological staining indicated that cells were able to uniformly distribute within the pores of hydrogels. Furthermore, the hydrogel did not hinder cells capability to survive and differentiate, nor did they exacerbate adaptive immune response, as histological staining showed no significant differences between the correspondent markers in NPC‐loaded hydrogel and control transplant (free NPC) groups (Figure 8b).^[^ ^134^ ^]^

As a result of the high modularity of granular hydrogels, the cell fate and function can be directed by adjusting the composition, size, shape, and porosity of packed microparticles. For example, human dermal fibroblasts were cultured on HA granular hydrogel composed of various diameter ranges of microparticles, from small (20–60 µm), medium (60–100 µm) to large (100–200 µm), and demonstrated significant dependence of cell ingrowth and proliferation on microparticle size. Cells showed lower proliferation and transgene expression in the small‐diameter microparticle group, while they adopted a more flattened shape and covered microparticles in the large‐diameter microparticle group (Figure 8c).^[^ ^49^ ^]^ Zenobi‐Wong's group encapsulated human primary chondrocytes in ZI granular hydrogels with 13–20% porosity and demonstrated higher cell proliferation and chondrogenesis in granular hydrogels with larger void space (150 µm‐grid microparticles vs 50 µm‐grid).^[^ ^13^ ^]^

Compartmentalization in granular hydrogels offers a high level of biological complexity by offering the capability of embedding multiple cell types. Feng et al. introduced a GelMA‐based biphasic granular bioprinted scaffold for the incorporation of two different cell types. Biphasic granular bioink has the ability to create a greater extent of microenvironment heterogeneity by incorporating different cell lines in each phase.^[^ ^122^ ^]^ Primary human hepatocytes were encapsulated within the microparticles, and endothelial cells (HUVEC) were incorporated within the interstitial hydrogel. The results proved the confinement of HUVEC to the interparticle region and exhibited a high proliferation rate with an elongated morphology. The authors attributed this observation to the enhanced cellular interactions and locally increased cell density for both cell lines.^[^ ^122^ ^]^

### 4.4 Granular Hydrogels as Cell‐Embedded Platforms for Spheroid Formation and Growth

Another emerging application of granular hydrogels is the formation and growth of organoids and spheroids. One of the main challenges in embedding spheroids within granular hydrogels is a change in interparticle crosslinking and reduction in their connectivity, due to the relatively high spheroid diameter, that might lead to construct collapse. Caprio et al. proposed a cartilage tissue regeneration approach based on granular composites composed of adult mesenchymal stem cells (MSCs) spheroids and NorHA microparticles, and the initial porosity of ≈20%.^[^ ^124^ ^]^ The group investigated the connectivity of both microparticles and spheroids components by calculating their initial volume ratio, which eventually impacts their stability and tissue formation. The proper volume ratio formulations were found to be 20:80 and 35:65 spheroid to microparticles, based on both the construct stability and their injectability. After 56 days of culture in chondrogenic medium, the volume ratio of 35:65 group showed significantly higher content of ECM, consisted of sGAG and COL, by ≈50%, which was likely due to the higher initial cell density. Regarding their stability, although the 20:80 group exhibited higher compressive modulus by ≈40% at d1, the 35:65 group increased compressive modulus greater by ≈30% than the 20:80 group at d56, due to the greater ECM deposition (Figure 8d).^[^ ^124^ ^]^

Alternatively, several studies have demonstrated the aggregation of cells and spheroid formation after culturing in granular hydrogels.^[^ ^135^, ^136^ ^]^ For example, Zhang et al. utilized a porous granular hydrogel as a platform to produce spheroids before employing them for extrusion 3D printing. This study developed a system based on ZI microparticles with average diameter of ≈15 µm, pore size of ≈200 µm, and porosity of ≈85%, through pore‐forming method. At 24 h post‐seeding adipose derived stem cells (ASCs) in granular hydrogel, cells tended to aggregate with the diameter of ≈110 µm, and performed good printing fidelity, when used as a spheroid‐laden bioink. High viability and structural stability of spheroids and their good stemness maintenance were confirmed immediately after printing and 7d after printing. In addition, when implanted into a cartilage defect in a rabbit knee joint model, the macroscopic and H&E staining images after 8 weeks revealed the coverage of defect with neo‐tissue and smooth integration with the host bone, as also confirmed with the higher deposition of cartilage matrix (GAGs and COL II).^[^ ^96^ ^]^

Thus, there is a great potential for further advancements in enhancing the physiological function of hydrogels and printed constructs, by understanding the effect of unique microporosity structure on spatiotemporal release of biological cues and tissue engineering stimulation. More in‐depth studies are needed to investigate the effect of mixing heterogenous cells with various cellular densities to recapitulate native organs. In addition, future work should explore the possibility of printing large 3D constructs with precise control over particle‐particle and particle‐cell interactions, as well as their orientation.

## 5 Conclusions and Future Perspectives

The microporosity, injectability, and modularity of granular hydrogels provide the potential to improve injectable biomaterials and bioprinted constructs for regenerative therapies. They have been able to improve cellular performance, provide a high level of spatial heterogeneity, induce immunomodulatory effect, and enhance in vivo integration over nanoporous bulk hydrogels.

This review deliberated the current state‐of‐the‐art on the application of granular hydrogels as a therapeutic carrier for the efficient delivery of small molecules, as a cell‐free system to stimulate endogenous tissue repair, as a scaffold for spheroid fabrication, encapsulation, and guiding their behavior, or as a cell carrier/in vitro cell culture model to promote cell spreading, proliferation, migration and differentiation. We also focused on recent advances in this area, promising outcomes and challenges, and mainly highlighted the relationship of structure‐property‐function.

Recent years have already seen the proper combinations of natural and synthetic biopolymers to compensate their respective weakness and leverage their strength. The principles of these hydrogelation chemistries are expected to give rise to advance material‐based properties of granular hydrogels. These advances alongside a vast library of available hydrogel designs will expand the design of more complex granular hydrogels. Specifically, in recent years, research on granular hydrogels has been directed to find ways to increase their multi‐responsiveness and multi‐functionality to allow for complex release and degradation profiles. Through modulating intra‐ and interparticle crosslinking, composition, combination of various types of microparticles, and addition of adhesive and signaling ligands, nanoparticles, and bioactive components, such as drugs and growth factors, tissue regeneration processes can be controlled in a more targeted manner.

Microparticles have proven to be effective tools for stimuli‐responsive release strategies into the environment, and thus, they can communicate through sensing and responding to various stimuli like pH, light, temperature, shear stress or a specific pathological stimulus. With the rapidly evolving field of chemistry plus improved stimuli‐responsive smart materials, we expect that the area of smart granular hydrogels will continue to grow. Possible directions in this area will likely explore flexible systems that respond to mechano‐chemo‐physical cues, as well as release molecules, dynamically adapt structure, or electrically stimulate cells to meet the complexities of a living environment. One of the exciting fields is to create multiscale properties to recapitulate the properties of native tissues and better understand how cells response to different microenvironments. For example, generating crosslinking density gradients in both individual and packed microparticles results in stiffness gradients.

Beyond the fabrication of granular hydrogels, in other fields granular hydrogels need further improvement, e.g. in the application of granular hydrogels in musculoskeletal tissue. In load‐bearing tissues faster disintegration of structure may occur compared to non‐granular hydrogels. The network gradients can be exploited to design a granular hydrogel that has a strain‐stiffening behavior for load‐bearing tissues, such as cartilage and tendon. Numerical simulations and computational models are invaluable tools that can be used to predict the materials properties and analyze their behavior in static states.

Still, the use of granular hydrogels for biomedical engineering field is in its early stages. For example, in the context of bioprinting, there is a lack of studies determining the stability of bioprinted granular constructs over long‐term incubation. Thus, more research is needed to explore new granular materials with improved mechanical integrity, especially for large‐scale constructs, and customizing complex cellular microenvironments.

Furthermore, to leverage the applicability of granular hydrogels for the next generation of scaffolds, more studies need to be done to tackle the challenges related to the generalizability of polymers and gelation methods for their fabrication, long‐term stability, larger animal models for in vivo studies, spatial signaling for cell differentiation, and computer simulations to optimize physicochemical properties of microparticles such as their size, shape, dispersity, and heterogeneity in obtaining optimized outcomes. It is important to consider the significant role of time dimension that is inherently coupled to degradability and stiffness and might complicate the analysis of structure‐function relationships.

As a new, just to be further explored field, a few studies took advantage of the heterogeneity of granular hydrogels, such as the incorporation of nanoparticles loaded in the voids among microparticles. For instance, by incorporating single or multiple nanoliposomes, nanogels, or nanovesicles within one hydrogel, more complex mechanical and cellular structures, that can more closely resemble the natural cellular microenvironment, can be achieved. Also, current applications of granular hydrogels are within the field of 3D cell‐laden or cell‐free biomaterials as a regenerative medicine strategy. Since there has been a growing interest in the application of microparticle hydrogels to generate giant unilamellar vesicles (GUVs) for artificial cell systems, it is envisaged that a similar approach can be exploited to generate assembled or annealed GUVs to find a key application in artificial cell technology development. Ultimately, with aqueous‐in‐aqueous compartments and cellular heterogeneity, efforts to investigate the potential of combinational therapies of granular hydrogels and therapeutics might be an attractive avenue in enhancing tissue regeneration.

## Conflict of Interest

The authors declare no conflict of interest.
